# Global hotspots of traded phylogenetic and functional diversity

**DOI:** 10.1038/s41586-023-06371-3

**Published:** 2023-07-26

**Authors:** Liam J. Hughes, Mike R. Massam, Oscar Morton, Felicity A. Edwards, Brett R. Scheffers, David P. Edwards

**Affiliations:** 1 Ecology and Evolutionary Biology, School of Biosciences, The University of Sheffield, Sheffield, UK; 2 Department of Wildlife Ecology and Conservation, Institute of Food and Agricultural Sciences, University of Florida, Gainesville, FL USA; 3 Present Address: RSPB Centre for Conservation Science, Cambridge, UK

**Keywords:** Conservation biology, Tropical ecology

## Abstract

Wildlife trade is a multibillion-dollar industry^1^ targeting a hyperdiversity of species^2^ and can contribute to major declines in abundance^3^. A key question is understanding the global hotspots of wildlife trade for phylogenetic (PD) and functional (FD) diversity, which underpin the conservation of evolutionary history^4^, ecological functions^5^ and ecosystem services benefiting humankind^6^. Using a global dataset of traded bird and mammal species, we identify that the highest levels of traded PD and FD are from tropical regions, where high numbers of evolutionary distinct and globally endangered species in trade occur. The standardized effect size (ses) of traded PD and FD also shows strong tropical epicentres, with additional hotspots of mammalian ses.PD in the eastern United States and ses.FD in Europe. Large-bodied, frugivorous and canopy-dwelling birds and large-bodied mammals are more likely to be traded whereas insectivorous birds and diurnally foraging mammals are less likely. Where trade drives localized extinctions^3^, our results suggest substantial losses of unique evolutionary lineages and functional traits, with possible cascading effects for communities and ecosystems^5,7^. Avoiding unsustainable exploitation and lost community integrity requires targeted conservation efforts, especially in hotspots of traded phylogenetic and functional diversity.

## Abstract (web summary)

Using a global dataset of traded species, it is found that the highest levels of traded phylogenetic and functional diversity are from tropical regions, where high numbers of evolutionary distinct and globally endangered species occur.

## Main

Wildlife trade is a multibillion-dollar industry^1^ encompassing over 100 million plants and animals traded annually^8^ as pets, food, traditional medicine and other products^9^. A quarter of terrestrial vertebrate species are traded^2^, along with thousands of invertebrate and plant species^10^. Exploitation is now a key driver of extinction risk^11^. The abundance of traded species declines on average by 62% where trade occurs^3^, rendering large areas of intact habitat denuded of its fauna^12^. Hotspots of trade species richness are primarily tropical, shaped by the underlying hyperdiversity of species^2^, high volumes traded in rural food markets for household consumption^13^ and strong international demand for wildlife, many as high-value pets or commodities^8,13^.

Conservation extends beyond the protection of species richness to include unique evolutionary histories^4^ and varied ecological roles^14^. Phylogenetic diversity (PD)—the cumulative evolutionary history of a set of species—and functional diversity (FD)—the diversity and distribution of functional traits within a set of species—are essential facets for biodiversity conservation (for example, The Intergovernmental Science-Policy Platform on Biodiversity and Ecosystem Services^11^) and increasingly inform conservation assessments^15^. By incorporating species evolutionary history (PD), morphology and ecological traits (FD)^4,16^, PD and FD are strong predictors of niche complementarity, ecological interactions^17^, resource-use efficiency^18^ and cascading effects for ecosystem functioning^5^, ecosystem services^6^ and resilience to disturbance^18^. For instance, overexploitation of large-bodied vertebrates disrupts seed-dispersal networks, impacting tropical forest tree communities and carbon stocking over time^19,20^. Trade also disproportionately targets evolutionarily distinct species—those isolated on an evolutionary tree—due to their rarity and/or unique features^2^, which can exaggerate impacts on communities and ecosystems.

We integrate evolutionary relationships and functional traits of 5,454 traded bird and mammal species to identify global epicentres of traded PD and FD, supporting more holistic and better-targeted conservation planning. The extent to which functional traits are phylogenetically conserved varies across taxa and regions^21^, and thus PD and FD should be used in tandem^15^. If conservation efforts are based solely on PD they may overlook regions where functional traits are weakly phylogenetically conserved, driving losses in ecosystem function. We thus extend our spatial analysis of trade richness to also include evolutionary distinctiveness and global endangerment (EDGE), and examine associations between trade and dietary and foraging traits with pronounced ramifications for ecological systems.

## Hotspots of traded PD and EDGE

### Phylogenetic diversity

**Fig. 1:**
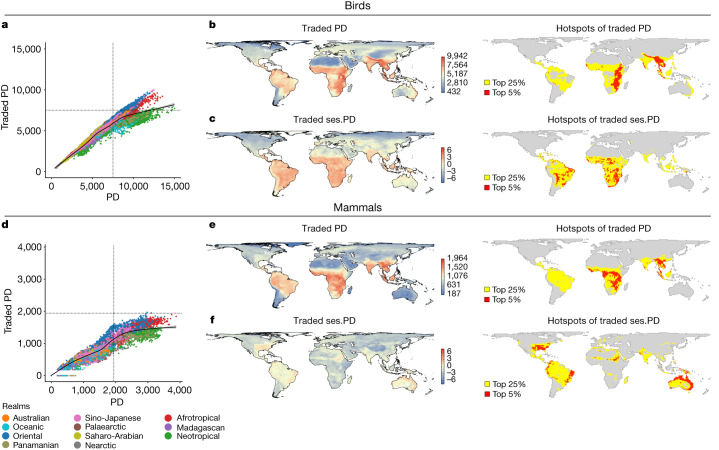
Levels of traded PD across the world for birds and mammals. **a**–**c**, Birds. **d**–**f**, Mammals. **b**,**e**, Map (**b**) and hotspots (**e**) of PD of traded species within each grid cell, with cells highlighted yellow representing the top 25% of grid cells and those in red representing the top 5%. **c**,**f**, Map (**c**) and hotspots (**f**) of traded ses.PD, with cells highlighted yellow representing the top 25% of grid cells and those in red representing the top 5%. **a**,**d**, Plots showing the relationship between traded PD and overall PD levels of each cell. Cells are colour coded by biogeographic realm. Black lines in scatterplots indicate locally estimated scatterplot smoothing (LOESS) fit (see Supplementary Fig. 2 for greater clarity on trends within each biogeographic realm). Units for PD denote the sum of all phylogenetic branch lengths.

Patterns of traded PD show hotspots largely in the tropical biogeographic realms of the Neotropics, Orient and Afrotropics (Fig. 1). Epicentres of traded PD (top 5% of cells) are concentrated within sub-Saharan Africa, the Western Ghats, mainland Southeast Asia and Sumatra (Fig. 1b,e). There is variation between taxa, with Himalaya and Ethiopian plateau epicentres for birds (Fig. 1b) and the Congo basin and Guinea forest epicentres for mammals (Fig. 1e). Epicentres of traded PD and traded species richness are similar^2^ (Supplementary Fig. 1), especially for mammals, which also includes the Western Ghats, whereas there are only a few epicentres of traded avian PD in the Neotropics but Tropical Andes and Guianan Shields are epicentres of traded species richness^2^.

We calculated a standardized effect size of traded PD (ses.PD) to identify where traded PD encompasses a broader phylogenetic breadth than expected given the species richness of the cells (that is, traded PD is overdispersed). Avian trade is more overdispersed than mammalian (ses.PD of 5.11 versus 2.68, respectively; Fig. 1c,f). Relative to epicentres of traded PD, those of traded avian ses.PD remain across sub-Saharan Africa and are gained in Neotropical dry forests and savannas (for example, Caatinga, Cerrado and Beni, Chaco) where trade spans phylogenetically distinct clades (Fig. 1c). The loss of hotspots from Asia probably reflects trade in many closely related species (for example, the Asian songbird crisis, where rampant demand for singing competitions has driven declines in many species^22^). Traded PD in mammals is more sensitive to species richness, with epicentres lost from across sub-Saharan Africa and Southeast Asia suggesting the targeting of phylogenetically young species (for example, antelope, deer). Epicentres were gained in the eastern United States, tropical Andes, Caatinga, Brazilian Atlantic, Saharan periphery and Australasia, which are not hotspots of traded species richness^2^. In the eastern United States the few traded species span widely different clades (for example, the bobcat *Lynx rufus*, coyote *Canis latrans*, American beaver *Castor canadensis* and northern racoon *Procyon lotor*), whereas in Australia hotspots are driven by ancient species including the short-beaked echidna *Ornithorhunchus anatinus*, which are occasionally seized in Southeast Asia^23^.

In regions of high traded PD, species from ancient lineages, often bearing unique traits (for example, the red-ivory casque of the helmeted hornbill *Rhinoplax vigil*^24^), are high-value commodities in urban and international markets^24,25^. High volumes of wildlife are also traded in many rural communities of sub-Saharan Africa and Southeast Asia^12^, where pervasive trade targets many species contributing to high traded PD. For example, 112 species have been recorded being kept as cagebirds in  Java, Indonesia^22^ and over 350 bird species, spanning 70 families, have been recorded in traditional medicine markets in sub-Saharan Africa^26^. Many Psitticadae (parrot) species are also widely traded internationally as pets^27^.

**Extended Data Fig. 1:**
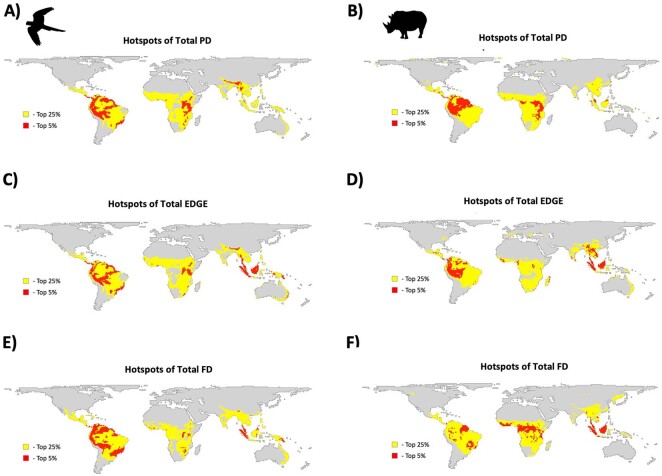
Global Hotspots per metric for all species. Hotspots of total PD for birds and mammals (A and B), hotspots of total EDGE for birds and mammals (C and D) and hotspots of total FD for birds and mammals. All species include both traded and untraded species. Yellow shaded areas being the top 25% hotspots and red areas being the top 5%.

Traded PD (Fig. 1b,e) is positively correlated with overall PD but varies by realm and taxa (Fig. 1a,d), showing new epicentres of overall PD in the Amazon and Brazilian Atlantic but none in Southeast Asia or in West and East (for birds) Africa (Extended Data Fig. 1a,b and Supplementary Tables 1 and 2). Biogeographical realms differ in the proportion of traded PD (birds, *χ*^2^ = 14,301, d.f. = 10, *P* < 0.001; mammals, *χ*^2^ = 1,652, d.f. = 10, *P* < 0.001; Extended Data Fig. 3a,b). The Neotropical realm has a lower proportion for both taxa versus other tropical realms (Extended Data Fig. 3a,b and Supplementary Table 3), and the lowest proportion of any realm for birds. Despite the Neotropics being a hotspot of traded avian species richness^2^, the lower proportion of overall PD suggests that trade occurs within a few highly speciose clades. For example, in Brazilian markets the recently radiated^28^ and speciose Emberizidae family dominates^29^. The lower proportion of overall PD traded in Neotropical mammals reflects the lower number of traded species^2^ and the recent radiation of many lineages relative to Old World mammals^30,31^.

Most avian PD is traded as pets whereas most mammalian PD is traded as products (Extended Data Fig. 3a,c). Levels of traded pet and product PD within cells are strongly associated, albeit with regional variation (Extended Data Fig. 3). For instance, traded avian PD in Australia is overwhelmingly comprised of pets but in the Palaearctic primarily of products, and a higher proportion of mammalian PD is traded as products in the Oriental and Afrotropical realms versus the Neotropical realm (Extended Data Fig. 3). These trends mirror patterns of traded species richness^2^, with large numbers of bird species in pet markets across the tropics^27,29^ and mammals dominating in food markets^32^. Mammalian PD in the pet trade is comparatively higher in the Neotropics (Extended Data Fig. 3d), with over 75% of Convention on International Trade in Endangered Species of Wild Fauna and Flora (CITES)-reported wild-caught primate and carnivore individuals exported from Latin America^33^.

Although hotspots of PD are identified using all traded species, some are not traded everywhere, risking commission-driven error. To account for this, we repeat our analysis of PD hotspots focusing only on realm-endemic species, removing species resident across realms (for example, the barn owl *Tyto alba*, tiger *Panthera tigris*) or migratory across realms (2023 (50.6%) avian and 322 (27.6%) mammalian species removed). The tropics remain the hotspot of endemic traded PD, with sub-Saharan Africa an epicentre (Extended Data Fig. 4a, b). Asian epicentres are lost (Extended Data Fig. 4a,b), because many widely trapped species have the majority of their distribution in the Oriental realm but also touch the Sino-Japanese realm (for example, the Asiatic black bear *Ursus thibetanus*, tiger and red-billed leiothrix *Leiothrix lutea*) or vice versa (the Chinese pangolin *Manis pentadactyla*). Similarly, many migratory bird species are heavily persecuted in South and Southeast Asia but not on their eastern Palaearctic breeding grounds.

### EDGE richness

**Fig. 2:**
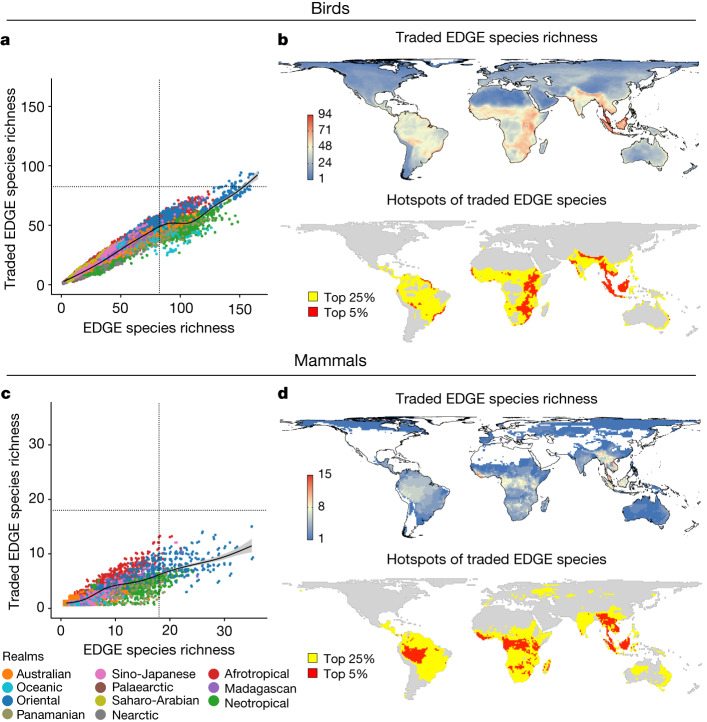
Species richness of the top 25% of traded EDGE species of birds and mammals. **a**,**b**, Birds (*n* = 997). **c**,**d**, Mammals (*n* = 270). **b**,**d**, Maps (**b**) and hotspots (**d**) of EDGE species richness. Cells highlighted yellow represent the top 25% of grid cells and those in red represent the top 5%. **a**,**c**, Relationship between species richness of the top 25% of traded species and the top 25% of overall species for each measure. Cells are colour coded by biogeographic realm. Black lines in scatterplots indicate a LOESS fit. Points jittered to avoid overlap in low EDGE species richness areas (see Supplementary Fig. 3 for greater clarity on trends within each biogeographic realm).

Trade in EDGE richness resembles patterns of PD, with the Oriental and Afrotropical realms broad hotspots (Figs. 2b,d and 1b,e). Re-analysis with only realm-endemic species again highlighted tropical hotspots of traded EDGE richness, with epicentres in sub-Saharan Africa and insular Southeast Asia (Extended Data Fig. 4c,d). Traded species in the Oriental and Afrotropical realms account for a higher proportion of the cumulative EDGE score (that is, EDGE summed across all species) than other tropical realms (Extended Data Figs. 3c,d and 5b,f and Supplementary Table 3), although maps of traded EDGE richness show unique epicentres in Western Amazonia and Borneo for both birds and mammals (Fig. 2b,d). Because a species with an extremely high EDGE value in a cell can eclipse many species with very low EDGE values, when using cumulative EDGE, we reran this analysis using log(mean EDGE). This shows new epicentres for both taxa in the Sahara, Horn of Africa, Madagascar, central Australia and Asia-Pacific Islands, plus the Middle East for birds (Extended Data Fig. 6).

Traded EDGE richness positively correlates with overall EDGE richness (Fig. 2a,c), with geographic and taxonomic variation (Extended Data Fig. 1c,d and Supplementary Tables 4 and 5). However, contrasting epicentres of traded EDGE richness (Fig. 2b,d) with overall EDGE richness (Extended Data Fig. 1c,d) indicates new epicentres of the latter in the Amazon (Eastern for mammals) and Andes, but reduced epicentres in sub-Saharan Africa and Indochina. Epicentres of traded EDGE richness are similar across the pet (dominated by birds; Extended Data Fig. 7c) and product (dominated by mammals; Extended Data Fig. 7g) trade.

EDGE captures species that are evolutionarily unique and threatened with extinction. Although trade correlates with threat status^2^, presence in trade does not necessarily mean that a species is threatened by trade^34^. Nevertheless, trade in threatened species often carries heightened extinction risk and can interact with deforestation and degradation in depletion of ecosystems of target species^35^. In Madagascar—an epicentre of traded and overall mammalian EDGE richness (Fig. 2d)—high levels of deforestation plus extraction for pets and bushmeat have led to the ancient Lemuriformes becoming highly threatened^36^.

EDGE overlooks evolutionarily distinct species that are not currently threatened, but may cause major losses of evolutionary history if locally overexploited. We repeated our analyses using evolutionary distinctiveness in trade. Relative to EDGE findings, this showed similar epicentres (Extended Data Fig. 9b,d), proportion of cumulative evolutionary distinctiveness traded between biogeographic regions (Extended Data Fig. 3e,f, Extended Data Fig. 5d,h and Supplementatry Table 3), log of mean evolutionary distinctiveness traded (Extended Data Fig. 6d,h) and correlations between evolutionary distinctiveness traded and overall richness (Extended Data Fig. 9a,c). Patterns for the pet and product trade were also similar (Extended Data Fig. 7c,g), except for in South America (Extended Data Fig. 7f) where many evolutionarily distinct species (for example, the kinkajou *Potos flavus* and brown-throated sloth *Bradypus variegatus*) are commonly traded as pets both domestically^32^ and internationally^33^.

## Hotspots of traded FD

**Fig. 3:**
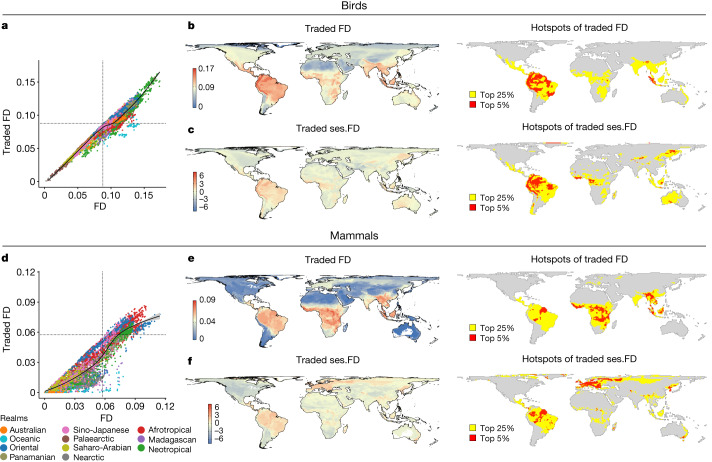
Levels of traded FD across the world for birds and mammals. **a**–**c**, Birds. **d**–**f**, Mammals. **b**,**e**, Maps and hotspots of FD of traded species within each grid cell, with cells highlighted yellow representing the top 25% of grid cells and those in red representing the top 5%. **c**,**f**, Maps and hotspots of traded ses.FD, with cells highlighted yellow representing the top 25% of grid cells and those in red representing the top 5%. **a**,**c**, Relationship between traded and overall FD levels of each cell. Cells are colour coded by biogeographic realm. Black lines in scatterplots indicate a LOESS fit (see Supplementary Fig. 4 for greater clarity on trends within each biogeographic realm).

Traded FD hotspots are predominantly pantropical (Fig. 3b,e). Epicentres of traded avian FD occur in tropical forests of insular Southeast Asia (Sundaland) and South America (Tropical Andes, Guianan Shields, Amazonia and Brazilian Atlantic), and in Neotropical savannas (Beni and Cerrado; Fig. 3b). Epicentres of traded mammalian FD again span Sundaland, Northeast Amazon and Beni, but not other Neotropical areas, instead including much of mainland Southeast Asia and sub-Saharan Africa (Fig. 3e). Although epicentres of traded FD and traded species richness have similarities^2^, there are key differences. Africa and mainland Asia are epicentres of traded avian richness but not traded FD, suggesting high functional redundancy, whereas the Northern Amazon and Brazilian Cerrado for birds and South America and Borneo for mammals are not epicentres of traded species richness^2^, pointing to trade in functionally unique species. Re-analysis with realm-endemic species reinforces the view that hotspots of traded FD are tropical, with epicentres remaining in Sundaland and sub-Saharan Africa (for mammals) (Supplementary Fig. 5e,f).

To identify where traded FD encompasses a broad range of traits independent of species richness (that is, traded FD is overdispersed), we calculated a standardized effect size of traded FD (ses.FD). Avian and mammalian trade are similarly overdispersed (Fig. 3c,f). Epicentres of traded avian ses.FD are still concentrated within the Neotropics, but also include East Asia, the Guinea forest and the Congo. Patterns of traded mammalian FD are more sensitive to species richness, with epicentres of ses.FD remaining in Northeast Amazon and Beni, lost from sub-Saharan Africa and Southeast Asia but gained in Western Amazon, Greenland, Europe and the Korean peninsula. Hotspots of ses.FD therefore occur in the biologically richest and poorest ecosystems. In the species-rich Amazon, traded species span a broad range of ecological functions including already-at-risk frugivores^37^ such as parrots, curassows, toucans and many Passerine songbirds (for example, tanagers^28^). In species-poor ecosystems, overexploitation of functionally unique species risks disproportionate effects on ecological function (for example, as seen in Europe).

**Extended Data Fig. 2:**
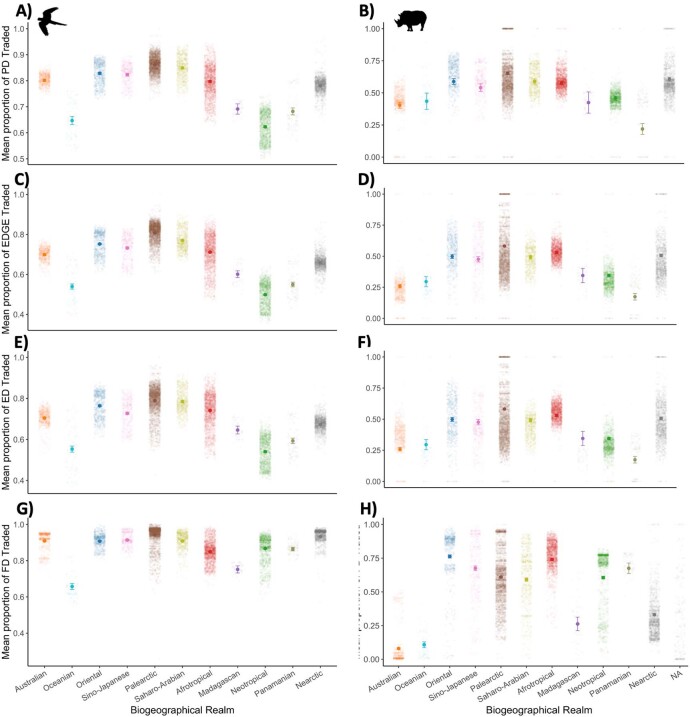
The mean proportion of each metric traded for birds and mammals between biogeographic realms. Beta regressions were used to determine whether the proportion of each metric traded differed by biogeographic realm for (A,C,E,G) birds and (B,D,F,H) mammals. (A and B) Phylogenetic Diversity- Birds: Pseudo R-squared= 0.3577, Log likelihood= 16240, df= 12, p < 0.05; Mammals: Pseudo R-squared= 0.1586; Log Likelihood 6776, df = 12, p < 0.05. (C and D) Summed Evolutionary Distinctiveness and Global Endangerment score- Birds: Pseudo R-squared= 0.3772, Log- likelihood= 12910, df= 12, p < 0.05; Mammals: Pseudo R-squared= 0.2189, Log-likelihood= 5551, df= 12, p < 0.05. (E and F) Summed Evolutionary Distinctiveness- - Birds: Pseudo R-squared= 0.2569, Log likelihood= 11880, df= 12, p < 0.05; Mammals: Pseudo R-squared= 0.1871, Log likelihood= 4685, df= 12, p < 0.05. (G and H) Functional Richness- Birds: Pseudo R-squared= 0.1902, Log-likelihood= 17880, df= 12, p < 0.05; Mammals: Pseudo R-squared= 0.5406, Log-likelihood= 5040, df= 12, p < 0.05.) Error bars represent 95% confidence intervals. Transparent points behind the means are the raw proportions of each grid cell. Biogeographic realms are colour coded.

**Extended Data Fig. 3:**
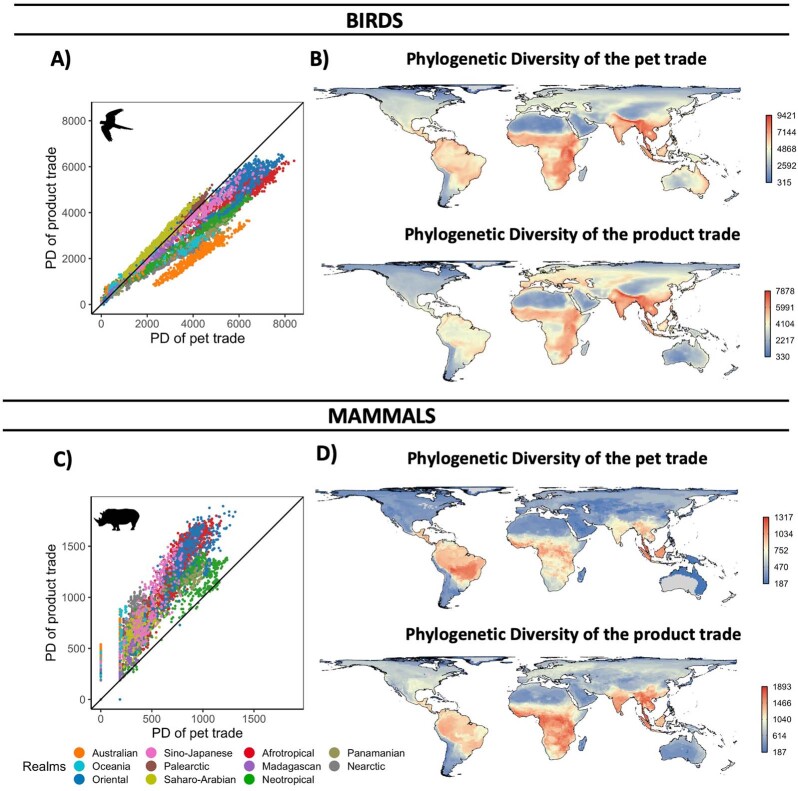
Pet and product traded PD for (A and B) birds and (C and D) mammals. Pet trade includes species traded as household pets, for expositions, circuses, or zoological gardens. Species traded for products include those used for bush meat, trophy hunting, clothing, medicine, or religious purposes. Points are color coded by the geographic realm. Points occurring above the 1:1 equivalency line indicate higher levels of trade as products than as pets.

**Extended Data Fig. 4:**
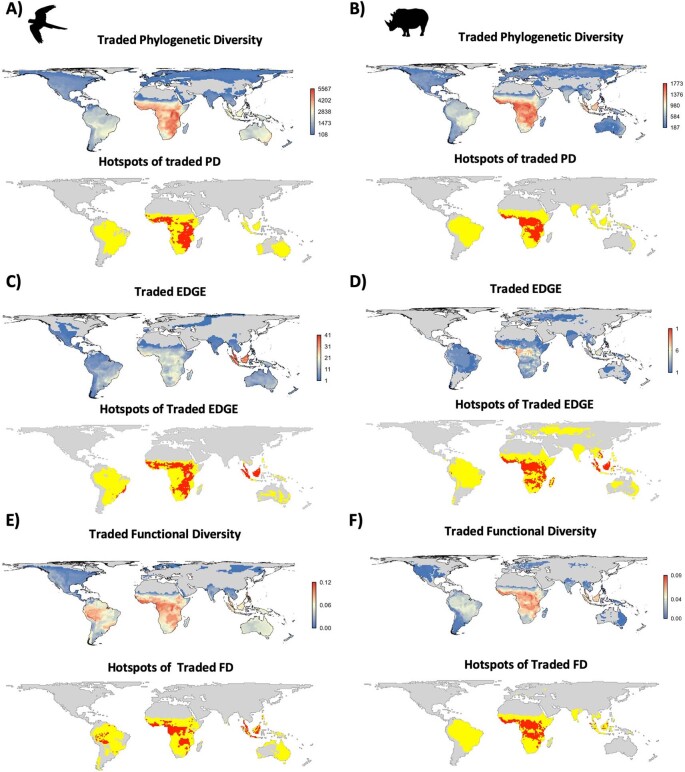
Traded PD (A and B), EDGE (C and D), and FD (E and F) of species endemic to biogeographic realms. Re-analysis of main text figures using only realm endemic species, see Methods for details. Cells highlighted as yellow represent the top 25% of grid cells, and those in red represent the top 5%.

**Extended Data Fig. 5:**
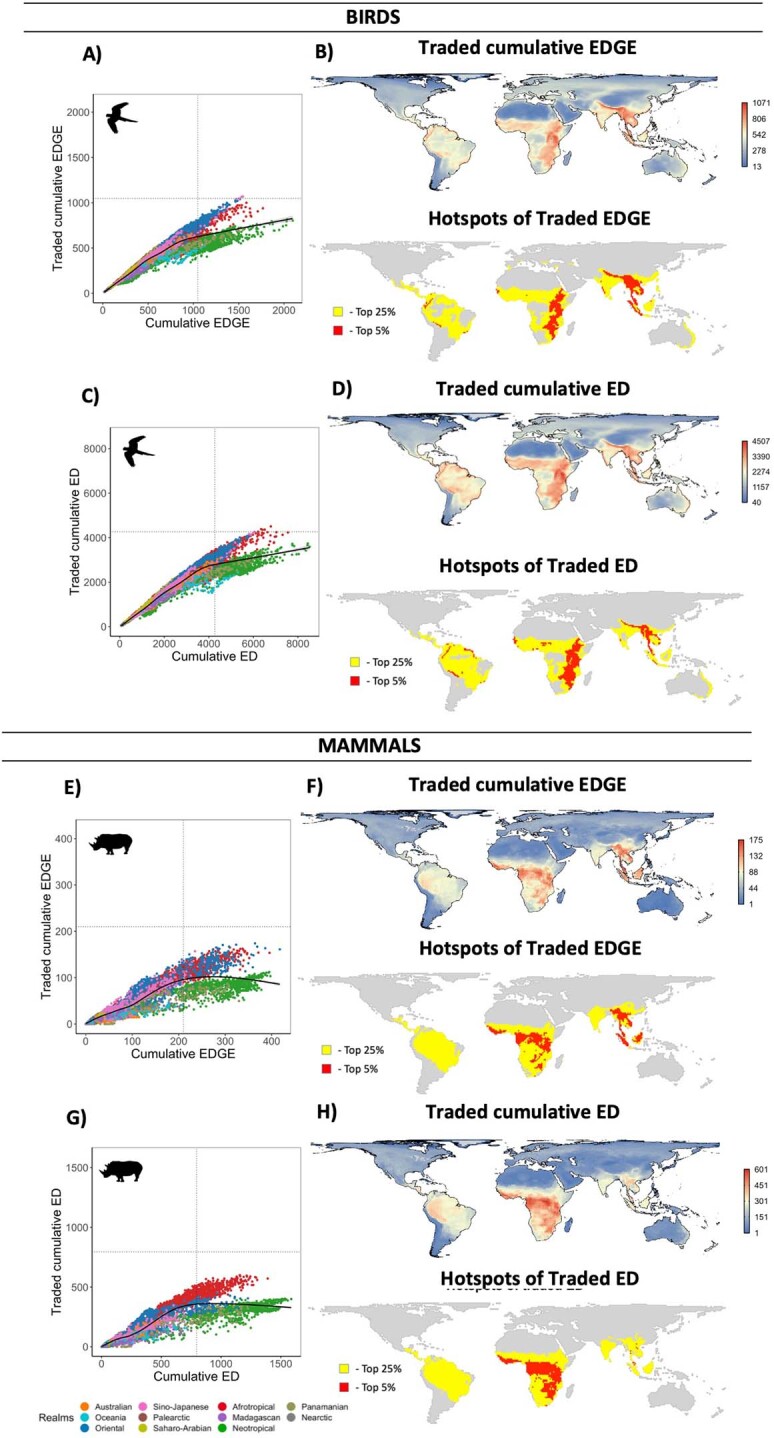
Maps and scatterplots of cumulative ED and EDGE. (A) and (E) cumulative ED and (C) and (G) cumulative EDGE for birds and mammals, respectively. (B), (D), (F) and (H) show the maps and hotspots of each measure. Cells highlighted as yellow representing the top 25% of grid cells, and those in red representing the top 5%. (A), (C), (E) and (G) show the relationship between the summed values of just traded species and the summed values of all species. Cells are colour coded by biogeographic realm. The black lines in scatterplots indicate a locally estimated scatterplot smoothing (LOESS) fit.

**Extended Data Fig. 6:**
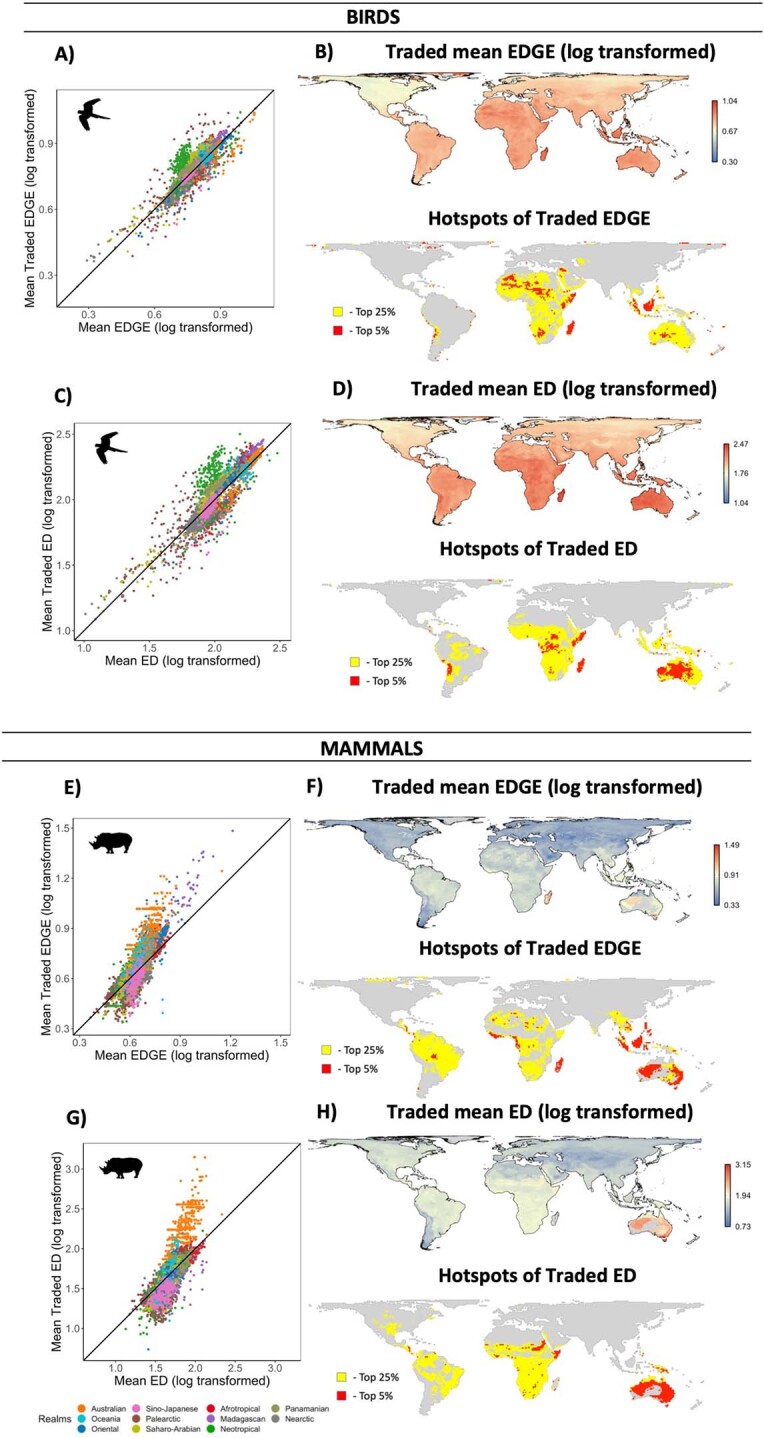
Maps and scatterplots of mean ED and EDGE (log transformed). (A) and (E) mean ED and (C) and (G) mean EDGE for birds and mammals, respectively. (B), (D), (F) and (H) show the maps and hotspots of each measure. Cells highlighted as yellow representing the top 25% of grid cells, and those in red representing the top 5%. (A), (C), (E) and (G) show the relationship between the summed values of just traded species and the summed values of all species Cells are colour coded by biogeographic realm. Points occurring above the 1:1 equivalency line indicate higher levels of trade as products than as pets.

Both traded and overall FD are positively correlated across taxa, although the association is weaker in some regions for mammals (Fig. 3a,d, Supplementary Fig. 4 and Suplementary Tables 6 and 7). Contrasting epicentres of traded FD (Fig. 3b,e) and overall FD (Extended Data Fig. 1e,f) show similar patterns for birds, whereas mammals have new epicentres of overall FD in the Brazilian Atlantic and Cerrado but none in Indochina and southern Africa where epicentres of traded FD were identified. Biogeographic realms differ in the proportion of FD traded for birds (*χ*^2^ = 5752.5, d.f. = 10, *P* < 0.001; Extended Data Fig. 2) and mammals (*χ*^2^ = 7764.5, d.f. = 10, *P* < 0.001; Extended Data Fig. 2). The Palaearctic realm has the highest proportion for birds, whereas the Oriental and Afrotropical then Panamanian and Sino-Japanese realms have the highest for mammals (Extended Data Fig. 2g,h and Supplementary Table 1). Most avian FD is traded as pets whereas most mammalian FD is traded as products (Extended Data Fig. 8a,c). Hotspots of FD in pet and product trade are similar between taxa (Extended Data Fig. 8b d), mirroring those for overall trade.

The exploitation of functionally diverse species from tropical forests and woodlands may disrupt the myriad of ecological functions provided by birds and mammals, with potential cascading community impacts and ramifications for ecosystem services^38,39^. Increased trade pressure reduces mammalian FD in Cameroonian forests^7^—an epicentre of traded and overall FD. In less speciose regions, trade may still have functional implications. Trade for pelts and food has contributed to substantial declines in the Mongolian marmot (*Marmota sibirica*)^40^, an ecosystem engineer whose colonies support greater abundances of steppe birds and mammals^41^.

## Dietary or foraging traits in trade

**Fig. 4:**
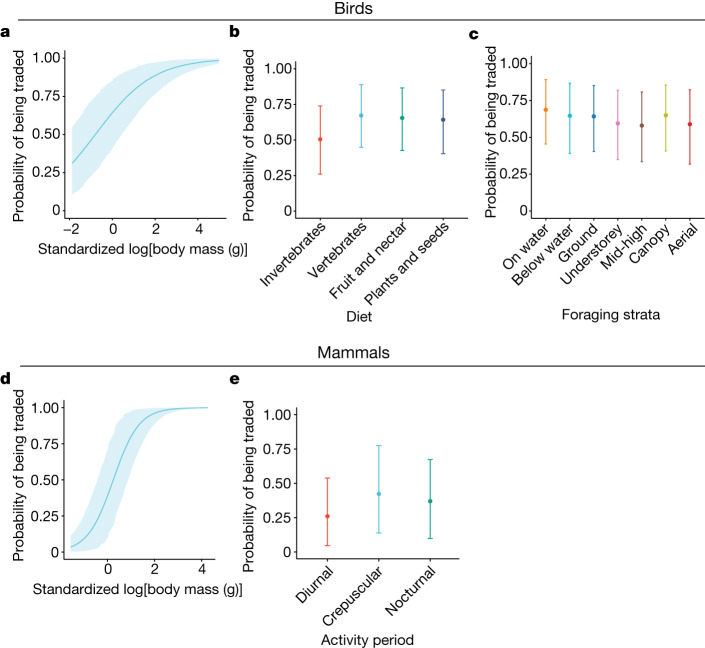
Probability of a species presence in trade across functional traits for birds and mammals. **a**–**c**, Birds. **d**,**e**, Mammals. **a**–**e**, Effect of standardized body mass (grams, log transformed (**a**,**d**)), avian diet (**b**), avian foraging strata (**c**) and mammalian activity period (**e**). Points (or line for body mass) represent median draw from the posterior. Confidence intervals represent the upper and lower 90% credible intervals. Draws from the posterior for a given trait were taken with all other traits set at the mean value for each respective taxonomic group. Birds, diet denotes plants and seeds; foraging strata denotes ground; activity period denotes not nocturnal; standardized body mass is 0; mammals, diet denotes plants and seeds; foraging strata denotes ground; activity period denotes nocturnal; standardized body mass is 0.

Body size has a clear positive association with a species’ probability of being traded (birds, 0.74, 90% credible interval 0.592–0.890, maximum probability of effect (MPE) = 100%; mammals, 1.832, 90% credible interval 1.61–2.06, MPE = 100%; Fig. 4a,d and Supplementary Table 8). Large-bodied species have increased detectability, higher value-per-unit hunting effort^13^ and are often considered attractive (for example, parrots)^20^, and many are particularly vulnerable to exploitation. Unregulated trade could downsize faunal communities, diminishing the key functional roles played by species^42^, including large seed dispersal, resulting in long-term shifts in tree communities and eroded carbon stores in tropical forests^19,20^.

Other functional traits show some association with presence in trade although effects are less pronounced (Fig. 4). Dietary, foraging and activity traits were dummy coded (as multiple binaries) rather than a single-factor variable to accommodate the fact that species can be fruit and seed eating, not fruit or seed eating. For birds, species that consume fruit are more likely to be traded than those that do not (0.25, 90% credible interval 0.07–0.42, MPE = 98.92%; Fig. 4b), potentially due to their ease of care as pets, with implications for long-distance seed dispersal that maintains forest diversity and, connectivity in fragmented landscapes^43^ and enhances plant diversity in recovering forests^44^. The consumption of insects is associated with a decreasing presence in bird trade (–0.37, 90% credible interval –0.54 to –0.21, MPE = 100%; Fig. 4b). The consumption of vertebrates (0.32, 90% credible interval 0.05–0.60, MPE = 97.06%) or of plants and seeds (0.18, 90% credible interval –0.01 to 0.36, MPE = 93.70%) slightly increases the probability of being traded, but the effect is uncertain (Fig. 4b). Canopy foraging was positively associated with trade presence (0.25, 90% credible interval 0.09–0.41, MPE = 99.29%; Fig. 4c), reflecting the prevalence of parrots, hornbills and tanagers in trade and suggesting disruption of ecological processes within tropical canopies. Species that forage terrestrially (0.21, 90% credible interval 0.04–0.39, MPE = 97.49%) or on water (0.43, 90% credible interval 0.06–0.79, MPE = 97.05%) may have a higher presence in trade than those that do not, although these effects are more uncertain. Activity period shows no influence on the likelihood of trade (Extended Data Fig. 10a).

For mammals, diurnality is associated with species being less likely to be traded (–0.592, 90% credible interval –1 to 0.17, MPE = 99.09%; Fig. 4e). Diurnal species are proportionally more prevalent in high-latitude regions^45^, where fewer species are traded^2^. Foraging strategy shows no influence on the likelihood of trade (Extended Data Fig. 10b).

## Conclusions

High levels of unique evolutionary lineages and functional diversity subject to trade across much of the tropics highlight the critical need for studies that directly assess the impact of exploitation on these facets of diversity^31^, especially at local and/or national scales^20^. There is a substantial risk that trade will drive major losses of evolutionary history and degrade ecosystem functioning. One report examining non-detriment findings for legal trade in CITES-listed species found that most assessments lacked consideration of offtake on ecosystem function^46^. This is especially important in the tropics, where forest disturbance has already reduced both the PD and FD of communities^15^, suggesting that poorly regulated trade could have compounding impacts. Pristine forests can be emptied of species via overharvesting^3,12,20^, and our findings suggest that poorly regulated trade in animal communities rich with PD and FD—including species from ancient lineages and functionally distinct groups—may reduce ecological functioning and ecosystem services in these threatened habitats^6,20^.

A multifaceted conservation approach, integrating community-based measures, traditional enforcement and demand-reduction campaigns^47^, is needed to lessen impacts in global hotspots of traded PD, EDGE richness and FD. Such efforts are essential to avoid unsustainable exploitation and ensure that trade does not result in the loss of unique evolutionary lineages and long-term state shifts of ecosystem functioning, with cascading effects for biodiversity and human communities. When focusing scarce conservation resources—both financial and political—it is important that the global epicentres of traded phylogenetic and functional diversity are considered.

## Methods

### Trade data

To determine whether a species was traded we used the global terrestrial vertebrate dataset compiled by Scheffers et al.^2^, filtering for birds and mammals. This contains extensive data from CITES and the International Union for Conservation of Nature (IUCN) red list. The IUCN list was generated via text-string search and manual reading to confirm trade. All species listed on CITES Appendix II as being ‘look-alike species’ and that are not traded themselves were considered as not traded in this study; eight species recently listed as extinct or extinct in the wild were removed. This resulted in a database of 4,265  avian and 1,189 mammalian species known to be traded from a total of 10,267 avian and 5,419 mammalian species (see Supplementary Table 9 for numbers of species used in each analysis). From this dataset we also extracted information on whether a species was traded as a product (that is, dead when used) or as a pet (that is, alive when used). A species can be traded as both a product and a pet.

### Spatial analyses

We divided the world into 111 × 111 km^2^ grid cells using a cylindrical equal-area projection and removing coastal cells consisting of less than 30% land. Species range maps were obtained from the IUCN Red List^48^ and superimposed onto this grid, with their presence/absence within each cell being recorded. A species was recorded as being present if its distribution overlapped any part of the cell. Each taxon was recorded as either traded or not (4,265 traded avian and 1,189 traded mammalian species). To compare geographical patterns in trade across biologically meaningful regions, we assigned each grid cell to one of the 11 biogeographical realms classified by Holt et al.^49^.

### Hotspots of traded PD and EDGE richness

In calculation of PD we used the most comprehensive global, time-calibrated species-level phylogenetic trees available. For birds this was derived from Jetz et al.^50^, overlaid on a Hackett family-level backbone containing 9,993 species; for mammals, the phylogenetic tree provided by Upham et al.^51^ containing 5,325 species. Nomenclature of species was standardized according to the corresponding phylogenies, resulting in phylogenetic analyses undertaken using 9,792 avian species and 5,325 mammalian. In total, 432 avian and 94 mammalian species were lost from the dataset used for phylogenetic analyses because they were not present in the phylogenetic trees.

To determine the hotspots of traded PD, Faith’s PD (from now on, PD, which is the sum of all branch lengths on a phylogenetic tree for a given set of species^4^), was calculated for each grid cell using the R package picante^52^. For birds and mammals, separately, this was calculated for all species within a grid cell followed by traded species only. To assess whether there are regional differences across different types of trade, PD was finally calculated for species traded as pets and those traded as products. To account for phylogenetic uncertainty, PD was calculated using 500 trees (for birds, 250 based on the Ericson backbone and 250 based on the Hackett backbone), with the median value for each grid cell being selected. Hotspot thresholds were set at the top 25 and 5% of grid cells, as per Scheffers et al^2^, to identify where trade is highest and to provide a measure of spread between these hotspots.

Because PD is correlated with species richness, to control for this and identify regions where a greater proportion of PD is traded than would be expected, we calculated ses.PD using the R package picante^52^. ses.PD compares the observed PD (PD_obs_) with that of null communities having the same species richness (equation (1)) to assess whether the observed PD is overdispersed (greater) or underdispersed (lower) in comparison with what would be expected under the null expectation of PD (PD_exp_) for a given community, and is calculated as

1 $$({\rm{Observed}}\,{\rm{PD}}-{\rm{mean}}\,{\rm{expected}}\,{\rm{PD}})/{\rm{SD}}({\rm{expected}}\,{\rm{PD}})$$

The expected PD values for each grid cell were determined by calculating the mean PD of 999 random null communities. Null communities were generated by randomization of species at the tips of the phylogeny but restricted to the regional pool of species present in the biogeographical realm of a given grid cell, giving geographically plausible null communities with species richness maintained. Due to the computational load of calculating ses.PD, the PD of null communities was calculated using the median value from 200 phylogenetic trees as opposed to 500 for other metrics (for birds, 100 based on the Ericson backbone and 100 based on the Hackett backbone). Hotspot thresholds were again set at the top 25 and 5% of grid cells.

For all avian and mammalian species, evolutionary distinctiveness was calculated using the ‘fair proportion’ method in the R package picante^52^. This method divides the value of each branch length of a phylogenetic tree by the number of species at its tip^53^. Evolutionary distinctiveness measures the isolation of a species on a phylogenetic tree, usually expressed in units of time (per million years)^53^. As with PD, to ensure that our results were robust to phylogenetic uncertainty, evolutionary distinctiveness values were calculated using 500 trees (for birds, 250 based on the Ericson backbone and 250 based on the Hackett backbone), with the median value for each species being selected. Using these evolutionary distinctiveness metrics, EDGE scores for all species in the phylogeny (excluding species listed as data deficient on the IUCN Red list) were calculated by weighting a species’ evolutionary distinctiveness by its IUCN threat status (equation (2)). We used the method proposed by Isaac et al.^53^, whereby global endangerment is a species IUCN Red list Category, weighted as follows: least concern, 0; near threatened, 1; vulnerable, 2; endangered, 3; critically endangered, 4. Data-deficient species were excluded (see Supplementary Table 9 for number of species used):

2 $${\rm{E}}{\rm{D}}{\rm{G}}{\rm{E}}=\log (1+{\rm{E}}{\rm{D}})+{\rm{G}}{\rm{E}}\times \log (2)$$

Following calculation of EDGE scores for all species, the values of each metric for traded species present within each grid cell were summed to measure the cumulative levels of EDGE traded within each cell. As with PD, this process was additionally undertaken for all species within each cell to allow for comparisons between the two.

**Extended Data Fig. 7:**
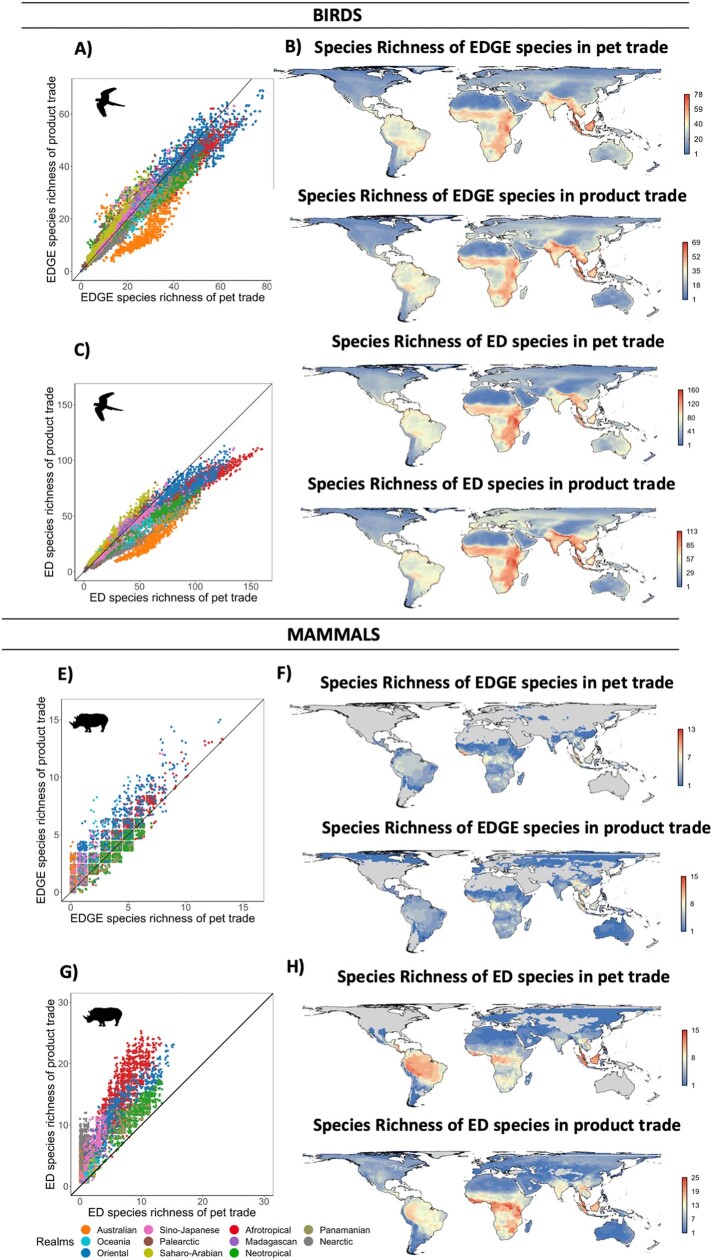
Pet and product trade of the top 25% of EDGE and ED species for (A-D) birds and (E-H) mammals. Pet trade includes species traded as household pets, for expositions, circuses, or zoological gardens. Species traded for products include those used for bush meat, trophy hunting, clothing, medicine, or religious purposes. Points are color coded by the geographic realm. Points occurring above the 1:1 equivalency line indicate higher levels of trade as products than as pets.

**Extended Data Fig. 8:**
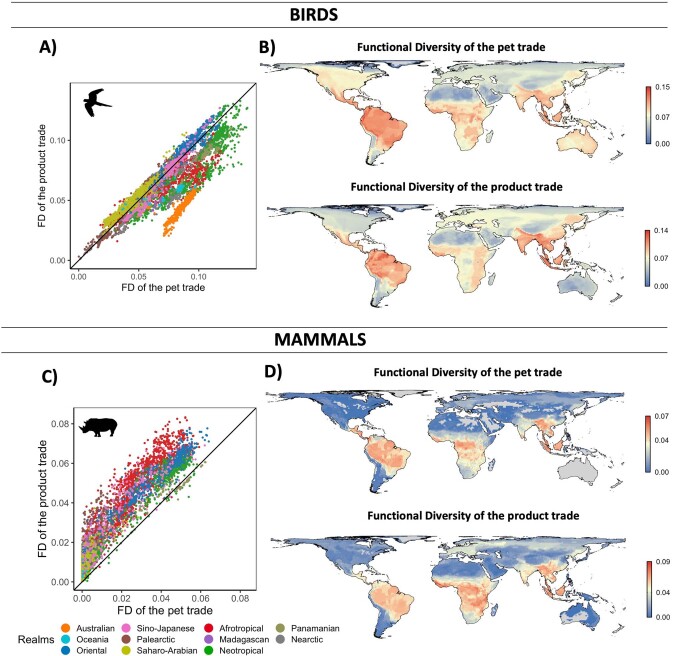
Pet and product traded FD. for (A and B) birds and (C and D) mammals. Pet trade includes species traded as household pets, for expositions, circuses, or zoological gardens. Species traded for products include those used for bush meat, trophy hunting, clothing, medicine, or religious purposes. Points are color coded by the geographic realm. Points occurring above the 1:1 equivalency line indicate higher levels of trade as products than as pets.

**Extended Data Fig. 9:**
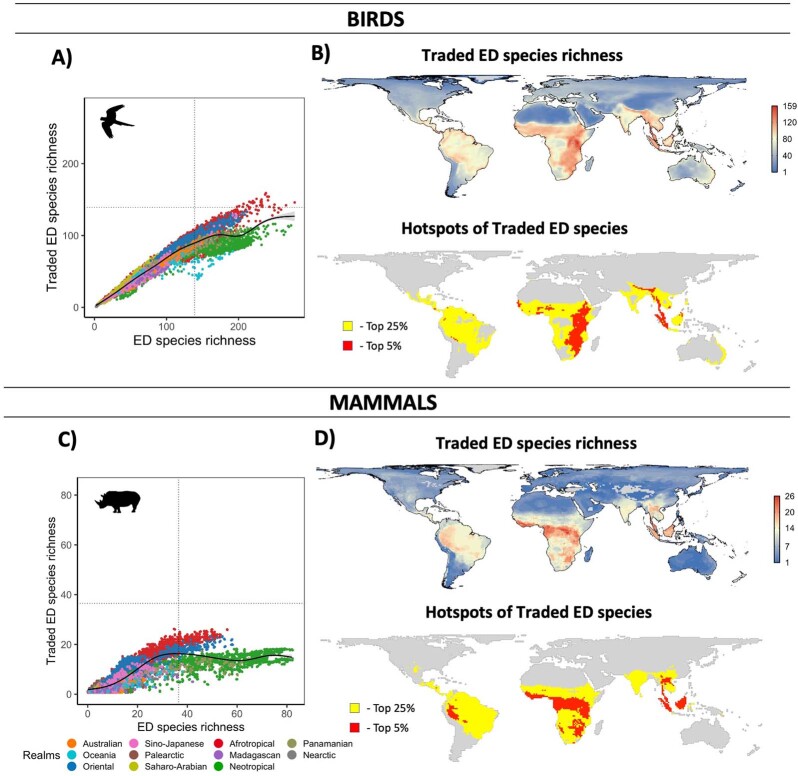
Species richness of the top 25% of traded evolutionary distinct species for (A) birds (n = 999) and (C) mammals (n = 277). (B), and (D) show the maps and hotspots of species richness. Cells highlighted as yellow representing the top 25% of grid cells, and those in red representing the top 5%. (A) and (C) show the relationship between the species richness of the top 25% of traded species and the top 25% of overall species for each measure. Cells are colour coded by biogeographic realm. The black lines in scatterplots indicate a locally estimated scatterplot smoothing (LOESS) fit.

**Extended Data Fig. 10:**
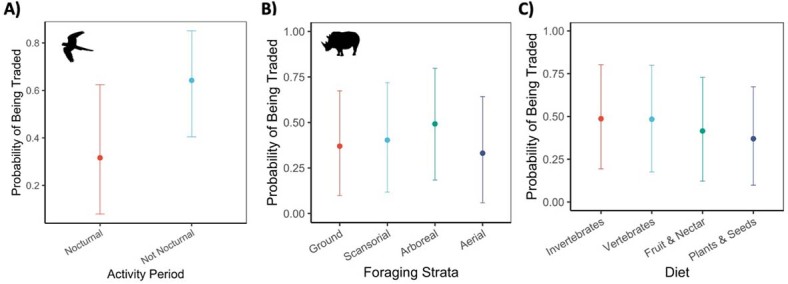
Probability of a species presence in trade across functional. traits for (A) Avian activity period, (B) Mammalian foraging strata, and (C) Mammalian diet. Points represent the median draw from the posterior. Confidence intervals represent the upper and lower 89% higher density confidence intervals (HDCI). Draws from the posterior for a given trait were taken with all other traits set at the mean value for each respective taxonomic group (Birds: Diet = plants and seeds, Foraging strata= Ground, Activity period= not nocturnal, standardized body mass= −0.227; Mammals: Diet = plants and seeds, Foraging strata= Ground, Activity period= Nocturnal, standardized body mass= −0.2507744).

The top 25% of EDGE traded species was then extracted (see Supplementary Table 9 for species numbers) and their range maps overlaid to calculate their species richness in each grid cell. This was done separately for birds and mammals, allowing identification of the regions with the highest richness of traded EDGE species. To determine whether these regions differ from overall species trends, this process was then repeated using the top 25% of all EDGE species (see Supplementary Table 7 for species numbers). We also generated richness maps for those traded as pets and those as products. Hotspot thresholds were again set at the top 25 and 5% of grid cells with the highest species richness levels. We repeated this process using species evolutionary distinctiveness, and present these results in Extended Data Fig. 7.

### Regional differences in proportion traded

Along with identification of hotspots for PD, evolutionary distinctiveness and EDGE, we also tested whether biogeographic realms differed in the proportion of each metric that was traded. To assess this, the proportions of PD, cumulative EDGE and cumulative evolutionary distinctiveness traded per grid cell were first calculated and then we fitted beta-regression models using the R package betareg^54^. Because our data did not fulfil the assumption that all values must fall between 0 and 1 (in some grid cells 0 and 100% of the community was traded), we transformed the proportions using the method proposed by Smithson and Verkuilen^55^ whereby *n* is sample size and *y* is the proportion of the respective measure subject to trade (equation (3)). The models were fit with biogeographic realm as the sole fixed effect. Model fit was assessed via diagnostic plots following Cribari-Neto and Zeilis^54^. Likelihood ratio tests were used to assess whether the effect of biogeographic realm was significant, followed by post hoc Tukey tests to evaluate differences between specific realms:

3 $$(\,y(n-1)+0.5)/n$$

### Hotspots of traded FD

Functional trait data for birds and mammals were extracted from Wilman et al.^56^ and assigned at species level. Four functional traits were used to calculate FD: (1) body mass (log transformed); (2) dietary composition; (3) foraging strata; and (4) activity period (details given in Supplementary Table 10). These traits cover a large proportion of Eltonian niche space^56^, providing information on multiple aspects of resource use and ecosystem interactions, such as the quantity and type of resources consumed by each species and where, within ecosystems, these interactions take place. They are thus representative of important functional dimensions of both birds and mammals.

Although all bird species within our phylogeny had trait data, 351 mammalian species present in the Upham phylogeny were missing trait data. For these species, missing traits were phylogenetically imputed using maximum-likelihood ancestral state reconstruction with the R package Rphylopars^57^ assuming Brownian motion. This imputation method increases the accuracy of the estimation of missing traits in comparison with other imputation approaches that do not consider phylogenetic information.

To ensure that this imputation process was appropriate we undertook the following steps. First we checked whether the traits showed a phylogenetic signal. For the two continuous traits (body mass and proportion of diet) we calculated Pagels lambda using the R package phytools^58^ and tested whether this was significantly different from the scenario in which the trait had evolved randomly. For the two discrete traits (foraging strata and activity period) we calculated the *D*-statistic using the R package caper^59^. The phylogenetic signal of all traits significantly differed than if the trait had evolved randomly (values presented in Supplementary Table 10).

Following this, we fitted three phylogenetic linear models (pGLMs) using the R package Rphylopars^57^. Each pGLM was fitted using using a different evolutionary model: Brownian motion, Pagel’s lambda, Ornstein–Beck and Kappa. When compared using the Akaike information criterion, the pGLM using a Brownian motion evolutionary model was found to fit our data best and this was the one selected to conduct our imputations. The categorical foraging stratum trait was dummy coded, setting ground, scansorial or aerial foraging to 1 for species that forage on each respective stratum. If imputed values for a species were 0 across all three foraging strategies, that species was set as arboreally foraging.

Given that simulations have shown that phylogenetic imputation is not always suitable even when phylogenetic signal is strong^60^, we then performed leave-one-out cross-validation on the pGLM to assess the accuracy of predictions. The results from this are presented in Supplementary Table 11. We evaluated the accuracy of our predictions using the mean absolute error and prediction coefficient as defined in ref. ^61^. Following this, all imputed traits were checked to ensure that they contained values consistent with a given trait type (for example, imputed dietary traits when rounded to the nearest 5 should sum to 100 to represent the proportion of a species diet). Through this, we identified the western sucker-footed bat (*Myzopoda schliemanni*) as having errors in its imputed trait values given that its predicted dietary traits summed to less than 10% of the overall proportion. Given this, we removed this species from our FD analyses. Finally we manually checked the imputed values of a random subset of 100 species to ensure that they were plausible given the information available on the species in the scientific literature. Following these processes, traits of 350 mammalian species were imputed and used in our functional analyses. This ultimately led to 15 avian and 81 mammalian species in our dataset being dropped from the FD analyses due to a lack of trait data and not being present in our phylogenies (Supplementary Table 9).

The metric used for FD was functional richness (from now on, FD), as described by Villéger et al.^62^, and has been used in similar global-scale analyses^21^. This index relies on placing a species within a multidimensional niche space in which the axes represent a combination of traits. FD quantifies the volume of this niche space occupied by the convex hull of a given set of species^62^. Higher FD values thus indicate a community having a wider range of trait values. FD relies on the assumption that species richness is greater than the number of traits, and thus cells with fewer than four species were removed. Because a combination of categorical and continuous traits was used, a pairwise species dissimilarity matrix of Gower distances was first calculated using the R package gawdis^63^, weighting traits so that each trait value contributed equally to the dissimilarity matrix. Principal coordinate analyses were then run, using three principal coordinate analyses axes, to gain the transformed coordinates, which were then used to calculate FD in the package mFD^64^. Hotspot thresholds were once again set at the top 25 and 5% of grid cells.

### Regional differences in proportion traded

Regional differences in the proportion of FD were also measured as above.

### ses.FD

Given that FD is also correlated with species richness^62^, to account for this we also calculated ses.FD of each grid cell following the same process as with ses.PD above.

### Precautionary re-analysis

Our primary analysis uses all species identified as being in trade to identify epicentres of traded PD, EDGE and FD. This approach reduces potential omission-driven errors because it captures all locations in which species may be traded. However, it has the potential to introduce commission-driven errors in the identification of epicentres by inclusion of species that are not actually traded across their entire range. This may be a particular problem for species with very large ranges that are traded across only a smaller portion of that range. We thus repeated all geographic analyses (PD, EDGE and FD), using only realm-endemic species, to substantially reduce the risk of commission-driven errors. The results of this are presented in full in Supplementary Information. However, we caveat that this re-analysis could introduce omission-driven discrepancies, in which species are traded across much of their range that spans realms or in which widespread species are traded in an area appropriately identified as an epicentre of trade (and not traded in areas that were not identified as epicentres) in our primary analysis, resulting in the loss of that epicentre.

### Prevalence of dietary or foraging traits in trade

Bayesian phylogenetic multilevel models were used to investigate whether any dietary or foraging-activity traits are associated with a species presence in trade. All species present in the global phylogenies were included in the models (9,835 avian and 5,325 mammalian species). We fit models using a Bernoulli distribution (logit link function) using the package brms^65,66^. Species presence in trade was the response variable, with each of the functional traits used in our functional diversity analyses (Supplementary Table 8) as explanatory variables (fixed effects). Dietary and functional traits represented as proportions were set as binary if they represented over 25% of a species’ diet/foraging stratum (Supplementary Table 4). Although body mass has already been identified as a key predictor of trade^2^, it was included as a fixed effect to account for correlations between size and other functional traits, and to assess whether the effect of body mass is still present when other functional traits are considered. log(Body mass) was standardized to have a mean of 0 and standard deviation of 1, to allow comparison with other traits in the model. Given that trade shows a phylogenetic signal^2^, the likelihood of a species being traded is non-independent and hence we computed a phylogenetic covariance matrix where the diagonal elements are equal to 1 using the R package ape^67^. The phylogenetic dependence of species was thus included as a random effect using this matrix.

Models were run with 4,000 iterations and 2,000 warm-up iterations in four Markov chains. All priors are zero-centred and diffuse to regularize parameter estimates and still explore plausible parameter space. A normal (0, 0.5) prior puts a priori weight on there being a 0.5 probability (inverse logit of 0) of the reference category being traded with a standard deviation of 0.5 on the logit scale. This incorporates almost the full range of values between 0 and 100% of being traded, without putting unnecessary weight on extremely high or low values: for example, an intercept prior centred on 3 on the logit scale would reflect the a priori expectation that over 95.2% of the reference category is traded. To ensure that chains were mixing and reached stable convergence, both models were visually assessed. Rhat (potential scale reduction values) values are below 1.02 for all model parameters, indicating convergence of both between- and within-chain estimates. Finally, to assess model adequacy, posterior predictive checks were undertaken using the predictive distribution in the R package Bayesplot^68^. These first compared our response variable with the simulated predictions from the model to ensure that the model had faithfully captured response distribution. We further checked the mean of the simulated data distribution with our response data to ensure that it was accurately recovered from the posterior. Finally we checked that no underlying patterns or discrepancies were present in the predictive error of predictive distribution.

For measures of uncertainty, the posterior distributions of each trait were summarized using medians and the 90% credible interval (highest density intervals). To assess the effect of functional traits, MPE estimates were computed for coefficients using the R package bayestestR^69^. MPE estimates—which range from 0.5 to 1.0—indicate the certainty of the direction of an effect and are generated from posterior distributions^69^. This index is highly correlated with the commonly used frequentist one- and two-sided *P* values and can therefore be useful for interpretation^70^. The MPE of parameters, alongside summaries of posterior distributions, was thus used to interpret the effect of having particular functional traits on a species’ likelihood of being traded. We assessed an MPE as being substantial where the probability of an effect going in a certain direction was over 97.50%. All analyses were undertaken using R v.4.2.1 (ref. ^71^).

### Reporting summary

Further information on research design is available in the Nature Portfolio Reporting Summary linked to this article.

## Online content

Any methods, additional references, Nature Portfolio reporting summaries, source data, extended data, supplementary information, acknowledgements, peer review information; details of author contributions and competing interests; and statements of data and code availability are available at 10.1038/s41586-023-06371-3.

## Supplementary information

Supplementary Information Supplementary Tables 1–11 and Figs. 1–4.

Reporting Summary

## Data Availability

Data used and produced by this study have been deposited in the University of Sheffield ORDA (Online Research Data) repository and is freely accessible at 10.15131/shef.data.23743848.

## Code availability

Code used in this study has been deposited in the University of Sheffield ORDA (Online Research Data) repository and is freely accessible at 10.15131/shef.data.23743848.
