# Supplementary material for: Global hotspots of traded phylogenetic and functional diversity
Source: Nature. 2023 Jul 26;620(7973):351–7. doi: 10.1038/s41586-023-06371-3 (PMC10412452; doi:10.1038/s41586-023-06371-3)
Supplement: Supplementary file 1 — Supplementary Tables 1–11 and Figs. 1–4. [file 41586_2023_6371_MOESM1_ESM.pdf]

nature portfolio

https://doi.org/10.1038/s41586-023-06371-3

Supplementary information

Global hotspots of traded phylogenetic and functional diversity

In the format provided by the authors and unedited

Supplementary materials

Global hotspots of traded phylogenetic and functional diversity

Liam J. Hughes[1], Mike R. Massam[1], Oscar Morton[1], Felicity A. Edwards[1]+, Brett R. Scheffers[2], David P. Edwards[1]*

[1]Ecology and Evolutionary Biology, School of Biosciences, The University of Sheffield, Sheffield, UK

[2]Department of Wildlife Ecology and Conservation, Institute of Food and Agricultural Sciences, University of Florida, Gainesville, FL, USA

*Correspondence: david.edwards@sheffield.ac.uk

+Present address: RSPB Centre for Conservation Science, David Attenborough Building, Pembroke Street, Cambridge CB2 3QZ, UK

Supplementary Table 1. Pearson correlation coefficient between traded PD and overall PD per realm for birds. Confidence intervals shown are 95% intervals and *n* denotes the number of grid cells per realm used in the analysis.

**Realm**	**Est.**	**Lower CI**	**Upper CI**	***p* value**	**t statistic**	***n***
Afrotropical	0.976	0.974	0.978	0.00000	196.407	1926
Australian	0.99	0.989	0.992	0.00000	179.495	645
Madagascan	0.958	0.926	0.977	0.00000	22.441	47
Nearctic	0.989	0.988	0.99	0.00000	250.699	1403
Neotropical	0.948	0.942	0.953	0.00000	112.236	1424
Oceania	0.868	0.796	0.915	0.00000	14.699	73
Oriental	0.928	0.916	0.938	0.00000	63.625	658
Palearctic	0.989	0.988	0.99	0.00000	362.187	2958
Panamanian	0.929	0.895	0.952	0.00000	24.25	96
Saharo-Arabian	0.991	0.989	0.992	0.00000	216.989	895
Sino-Japanese	0.984	0.98	0.987	0.00000	111.301	415

Supplementary Table 2. Pearson correlation coefficient between traded PD and overall PD per realm for mammals. Confidence intervals shown are 95% intervals and *n* denotes the number of grid cells per realm used in the analysis.

**Realm**	**Est**	**Lower CI**	**Upper CI**	***p* value**	**t statistic**	***n***
Afrotropical	0.969	0.966	0.972	0.00000	171.808	1926
Australian	0.841	0.816	0.862	0.00000	38.628	637
Madagascan	0.817	0.69	0.895	0.00000	9.383	47
Nearctic	0.76	0.737	0.781	0.00000	43.742	1403
Neotropical	0.95	0.945	0.955	0.00000	114.775	1422
Oceania	0.822	0.73	0.885	0.00000	12.154	73
Oriental	0.802	0.773	0.828	0.00000	34.385	658
Palearctic	0.837	0.826	0.847	0.00000	83.144	2962
Panamanian	0.935	0.902	0.957	0.00000	24.415	96
Saharo-Arabian	0.872	0.856	0.887	0.00000	53.301	894
Sino-Japanese	0.908	0.89	0.924	0.00000	44.123	415

Supplementary Table 3. Results from post-hoc Tukey test comparing whether biogeographic realms differ in the proportion of the respective metric traded. Groups indicate which realms significantly differ from each other (p<0.05).

Biogeographic Realm	Mean	Standard Error	Group	Mean	Standard Error	Group
		Birds			*Mammals*	
Phylogenetic Diversity						
Afrotropical	0.797	0.001	e	0.577	0.005	de
Australian	0.802	0.002	e	0.404	0.008	b
Madagascan	0.691	0.007	c	0.425	0.029	bc
Nearctic	0.780	0.001	d	0.606	0.005	f
Neotropical	0.623	0.001	a	0.458	0.006	c
Oceania	0.647	0.005	b	0.435	0.022	bc
Oriental	0.829	0.002	f	0.588	0.008	ef
Palearctic	0.861	0.001	h	0.652	0.004	g
Panamanian	0.682	0.005	c	0.219	0.015	a
Saharo-Arabian	0.849	0.001	g	0.589	0.007	ef
Sino-Japanese	0.823	0.002	f	0.541	0.010	d
Cumulative EDGE						
Afrotropical	0.712	0.001	f	0.530	0.005	e
Australian	0.699	0.002	e	0.259	0.007	b
Madagascan	0.601	0.009	c	0.345	0.029	bc
Nearctic	0.658	0.002	d	0.506	0.006	de
Neotropical	0.499	0.002	a	0.344	0.006	c
Oceania	0.539	0.007	b	0.295	0.021	bc

Oriental	0.752	0.002	h	0.498	0.009	de
Palearctic	0.808	0.001	j	0.582	0.004	f
Panamanian	0.550	0.006	b	0.174	0.013	a
Saharo-Arabian	0.770	0.002	i	0.492	0.008	d
Sino-Japanese	0.732	0.003	g	0.476	0.011	d
Cumulative ED						
Afrotropical	0.741	0.001	f	0.530	0.005	e
Australian	0.705	0.003	d	0.259	0.007	b
Madagascan	0.646	0.010	c	0.345	0.029	bc
Nearctic	0.673	0.002	c	0.506	0.006	de
Neotropical	0.540	0.002	a	0.344	0.006	c
Oceania	0.553	0.008	a	0.295	0.021	bc
Oriental	0.764	0.002	g	0.498	0.009	de
Palearctic	0.790	0.001	h	0.582	0.004	f
Panamanian	0.593	0.007	b	0.174	0.013	a
Saharo-Arabian	0.785	0.002	h	0.492	0.008	d
Sino-Japanese	0.727	0.003	e	0.476	0.011	d
Functional Diversity						
Afrotropical	0.850	0.001	c	0.741	0.004	f
Australian	0.909	0.002	e	0.080	0.003	a
Madagascan	0.752	0.010	b	0.262	0.026	b
Nearctic	0.933	0.001	f	0.330	0.005	b
Neotropical	0.867	0.001	d	0.610	0.005	c
Oceania	0.658	0.008	a	0.108	0.011	a

Oriental	0.908	0.002	e	0.763	0.006	f
Palearctic	0.943	0.001	g	0.610	0.003	cd
Panamanian	0.864	0.005	cd	0.675	0.020	de
Saharo-Arabian	0.909	0.002	e	0.591	0.006	c
Sino-Japanese	0.915	0.002	e	0.675	0.009	e

Supplementary Table 4. Pearson correlation coefficient between the top 25% traded EDGE species richness and overall EDGE richness per realm for birds. Confidence intervals shown are 95% intervals and *n* denotes the number of grid cells per realm used in the analysis.

**Realm**	**Est.**	**Lower CI**	**Upper CI**	***p* value**	**t statistic**	***n***
Afrotropical	0.974	0.972	0.976	0	190.595	1966
Australian	0.956	0.949	0.962	0	85.107	679
Madagascan	0.981	0.968	0.989	0	36.628	54
Nearctic	0.949	0.943	0.953	0	118.363	1569
Neotropical	0.955	0.951	0.96	0	123.92	1468
Oceanian	0.871	0.812	0.913	0	17.117	95
Oriental	0.959	0.953	0.964	0	91.001	730
Palearctic	0.956	0.953	0.959	0	187.059	3357
Panamanian	0.98	0.971	0.986	0	52.983	119
Saharo-Arabian	0.98	0.978	0.983	0	152.246	936
Sino-Japanese	0.977	0.972	0.981	0	95.721	436

Supplementary Table 5. Pearson correlation coefficient between the top 25% traded EDGE species richness and overall EDGE richness per realm for mammals. Confidence intervals shown are 95% intervals and *n* denotes the number of grid cells per realm used in the analysis.

Realm	Est.	Lower CI	Upper CI	*p* value	t statistic	*n*
Afrotropical	0.918	0.91	0.925	0	95.242	1966
Australian	0.632	0.583	0.677	0	20.422	679
Madagascan	0.889	0.813	0.935	0	13.718	54
Nearctic	0.428	0.353	0.498	0	10.464	1569
Neotropical	0.807	0.787	0.826	0	47.619	1468
Oceanian	0.586	0.343	0.755	0	4.569	95
Oriental	0.886	0.868	0.902	0	48.332	730
Palearctic	0.526	0.491	0.559	0	26.05	3357
Panamanian	0.564	0.393	0.697	0	6.033	119
Saharo-Arabian	0.199	-0.112	0.474	0.207583	1.281	936
Sino-Japanese	0.863	0.826	0.892	0	26.031	436

Supplementary Table 6. Pearson correlation coefficient between traded FD species richness and overall FD per realm for birds. Confidence intervals shown are 95% intervals and *n* denotes the number of grid cells per realm used in the analysis.

Realm	Est.	Lower CI	Upper CI	*p* value	t statistic	*n*
Afrotropical	0.971	0.968	0.973	0	178.589	1966
Australian	0.975	0.971	0.979	0	114.946	679
Madagascan	0.995	0.991	0.997	0	69.535	54
Nearctic	0.993	0.993	0.994	0	337.733	1562
Neotropical	0.979	0.977	0.981	0	185.801	1467
Oceanian	0.978	0.967	0.985	0	45.111	95
Oriental	0.989	0.987	0.99	0	177.879	730
Palearctic	0.996	0.996	0.997	0	659.633	3274
Panamanian	0.987	0.981	0.991	0	66.516	119
Saharo-Arabian	0.981	0.979	0.984	0	155.86	936
Sino-Japanese	0.993	0.992	0.994	0	176.849	436

Supplementary Table 7. Pearson correlation coefficient between traded FD species richness and overall FD per realm for mammals. Confidence intervals shown are 95% intervals and *n* denotes the number of grid cells per realm used in the analysis.

Realm	Est.	Lower CI	Upper CI	*p* value	t statistic	*n*
Afrotropical	0.975	0.972	0.977	0	190.947	1935
Australian	0.542	0.474	0.604	0	13.829	461
Madagascan	0.579	0.33	0.752	0	4.432	41
Nearctic	0.441	0.398	0.481	0	18.797	1469
Neotropical	0.98	0.978	0.982	0	184.925	1403
Oceanian	0.537	0.351	0.682	0	5.398	74
Oriental	0.82	0.794	0.843	0	37.228	676
Palearctic	0.822	0.81	0.833	0	79.653	3049
Panamanian	0.804	0.711	0.87	0	12.029	81
Saharo-Arabian	0.88	0.864	0.894	0	55.398	897
Sino-Japanese	0.9	0.88	0.917	0	42.033	418

Supplementary Table 8 - Model coefficients of the fixed effects included in the phylogenetic Bayesian models. Intervals represent the upper and lower bounds of the 90% highest density intervals. Values highlighted in bold are those where the HDCI does not cross 0 and the MPE is greater than 97.5%.

	Estimate	Lower	Upper	MPE (%)
**Birds**				
**Intercept**	-1.16	-2.58	0.30	
**Body Size**				
Body mass (log transformed)	0.74	**0.59**	**0.89**	100.00
**Diet**				
Invertebrates	-0.37	**-0.54**	**-0.21**	**100.00**
Vertebrates	0.32	**0.05**	**0.60**	**97.06**
Fruit & nectar	0.25	**0.07**	**0.42**	**98.92**
Plants & seeds	0.18	**-0.01**	**0.36**	**93.70**
**Foraging strata**				
Below water's surface	0.23	-0.25	0.70	78.24
On water's surface	0.43	0.06	0.79	97.05
Ground	0.21	0.04	0.39	97.49
Understory	0.01	-0.11	0.14	56.96
Mid-level canopy (>2m above ground)	-0.05	-0.20	0.10	72.10
Canopy	0.25	**0.09**	**0.41**	**99.29**
Aerial	-0.01	**-0.49**	**0.44**	**52.31**
**Activity period**				
Nocturnal	1.36	**0.31**	**2.48**	**97.75**
**Mammals**				

**Intercept**	-0.21	-1.88	1.48	
**Body Size**				
Body mass (log transformed)	1.83	**1.61**	**2.06**	**100.00%**
**Diet**				
Invertebrates	0.05	-0.32	0.43	59.63%
Vertebrates	0.04	-0.44	0.51	55.14%
Fruit & nectar	-0.24	-0.52	0.06	90.89%
Plants & seeds	-0.43	-0.83	-0.02	95.88%
**Foraging strata**				
Ground	0.19	-0.55	0.94	66.48%
Scansorial	0.33	-0.45	1.12	75.80%
Arboreal	0.69	-0.06	1.43	93.59%
**Activity period**				
Nocturnal	-0.08	-0.55	0.39	61.65%
Crepuscular	0.14	-0.20	0.47	75.48%
Diurnal	-0.59	**-1.00**	**-0.17**	**99.09%**

Supplementary Table 9 - Number of species in each analyses. The maximum number of species were selected based upon the availability of phylogenetic and functional trait data.

**Birds**		
	Traded species	All species
Phylogenetic Diversity	3995	9835
Top 25% of evolutionary distinct species	999	2458
Top 25% of EDGE species	997	2432
Functional Diversity	4257	10252
Cumulative ED	3995	9835
Cumulative EDGE	3989	9749
PD of species traded as products	2168	-
PD of species traded as pets	3440	-
Top 25% of evolutionary distinct species traded as products	622	-
Top 25% of evolutionary distinct species traded as pets	818	-
Top 25% of EDGE species traded as products	716	-
Top 25% of EDGE species traded as pets	736	-
FD of species traded as products	2280	-
FD of species traded as products	3678	-
**Mammals**		
Phylogenetic Diversity	1167	5325
Top 25% of evolutionary distinct species	292	1331
Top 25% of EDGE species	283	1163
Functional Diversity	1175	5338
Cumulative ED	1167	5325

Cumulative EDGE	1130	4652
PD of species traded as products	1055	-
PD of species traded as pets	1101	-
Top 25% of evolutionary distinct species traded as products	271	-
Top 25% of evolutionary distinct species traded as pets	111	-
Top 25% of EDGE species traded as products	263	-
Top 25% of EDGE species traded as pets	149	-
FD of species traded as products	1109	-
FD of species traded as pets	501	-

Supplementary Table 10 - Functional traits used in functional diversity analyses and their phylogenetic signal (for mammals). Traits denoted with a * were transformed to binary variables (if proportion was over 25% variable was set to 1) for the phylogenetic multilevel Bayesian models to aid model fit. Traits denoted with a † were standardized for phylogenetic multilevel Bayesian models. For diet and body size, phylogenetic signal shows the Pagels lambda value for the given trait, where 1 indicates it is perfectly phylogenetically conserved under an Brownian motion evolutionary model. For activity period and foraging stratum, phylogenetic signal shows the traits D-statistic whereby 0 represents a trait being perfectly conserved under a Brownian model of evolution. P-values represent the likelihood of whether the observed values would have occurred if traits had evolved randomly; P-values denoted + represent phylogenetic structure evolved under brownian motion.

**Functional Trait Type**	**Functional Trait**	**Variable Type**	**Phylogenetic Signal**	**P-value**
Mammals				
Diet	Insects	Proportion*	0.997	0
	Vertebrates	Proportion*	0.995	0
	Fruit & Nectar	Proportion*	0.993	0
	Plants & Seeds	Proportion*	0.993	0
Foraging Strata	Ground	Categorical	-0.152	0; 0.989+
	Scansorial	Categorical	0.100	0; 0.179+
	Arboreal	Categorical	-0.144	0; 0.979+
	Aerial	Categorical	-0.286	0; 1+
Activity Period	Diurnal	Binary	-0.039	0; 0.762+
	Crepuscular	Binary	0.020	0; 0.387+
	Nocturnal	Binary	-0.095	0; 0.911+
Species size	Body mass (in grams) (log transformed) †	Continuous	0.999	0

Supplementary Table 11 - Mean absolute error from leave-one-out cross validation of the imputed traits using the ML Ancestral State Reconstruction. The prediction coefficient (p^2) interpretation is as follows, 1 indicates a perfect match between prediction and observation and values approaching 0 (or negative) indicate a prediction no better than chance.

Functional Trait Type	Functional Trait	Mean Absolute Error	p^2
**Diet**	Invertebrate	1.003	0.939
	Vertebrate	-0.225	0.880
	Fruit & Nectar	-0.171	0.904
	Plants & Seeds	-0.628	0.945
**Foraging Stratum**	Ground	-0.011	0.905
	Scansorial	-0.002	0.611
	Aerial	0.007	0.962
**Activity Period**	Nocturnal	0.007	0.841
	Crepuscular	-0.001	0.750
	Diurnal	-0.006	0.813
**Body Size**	Body Mass (grams)	174.497	0.727

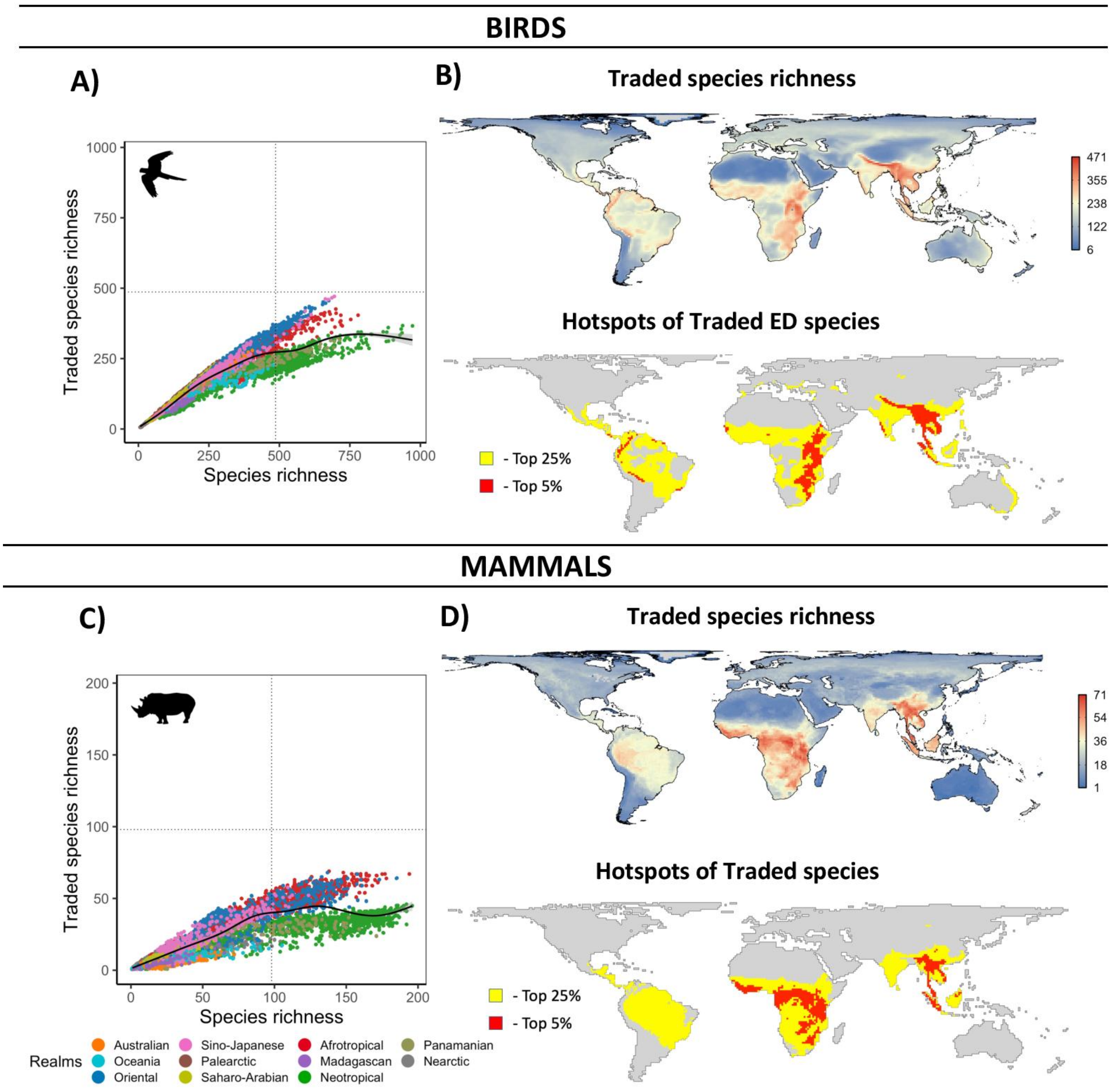

Figure S1- Richness of species in trade for (A) birds (n= 4265) and (C) mammals (n=1189). (B), and (D) show the maps and hotspots of species richness. Cells highlighted as yellow representing the top 25% of grid cells, and those in red representing the top 5%. (A) and (C) show the relationship between the species richness of the top 25% of traded species and the top 25% of overall species for each measure. Cells are colour coded by biogeographic realm. The black lines in scatterplots indicate a locally estimated scatterplot smoothing (LOESS) fit.

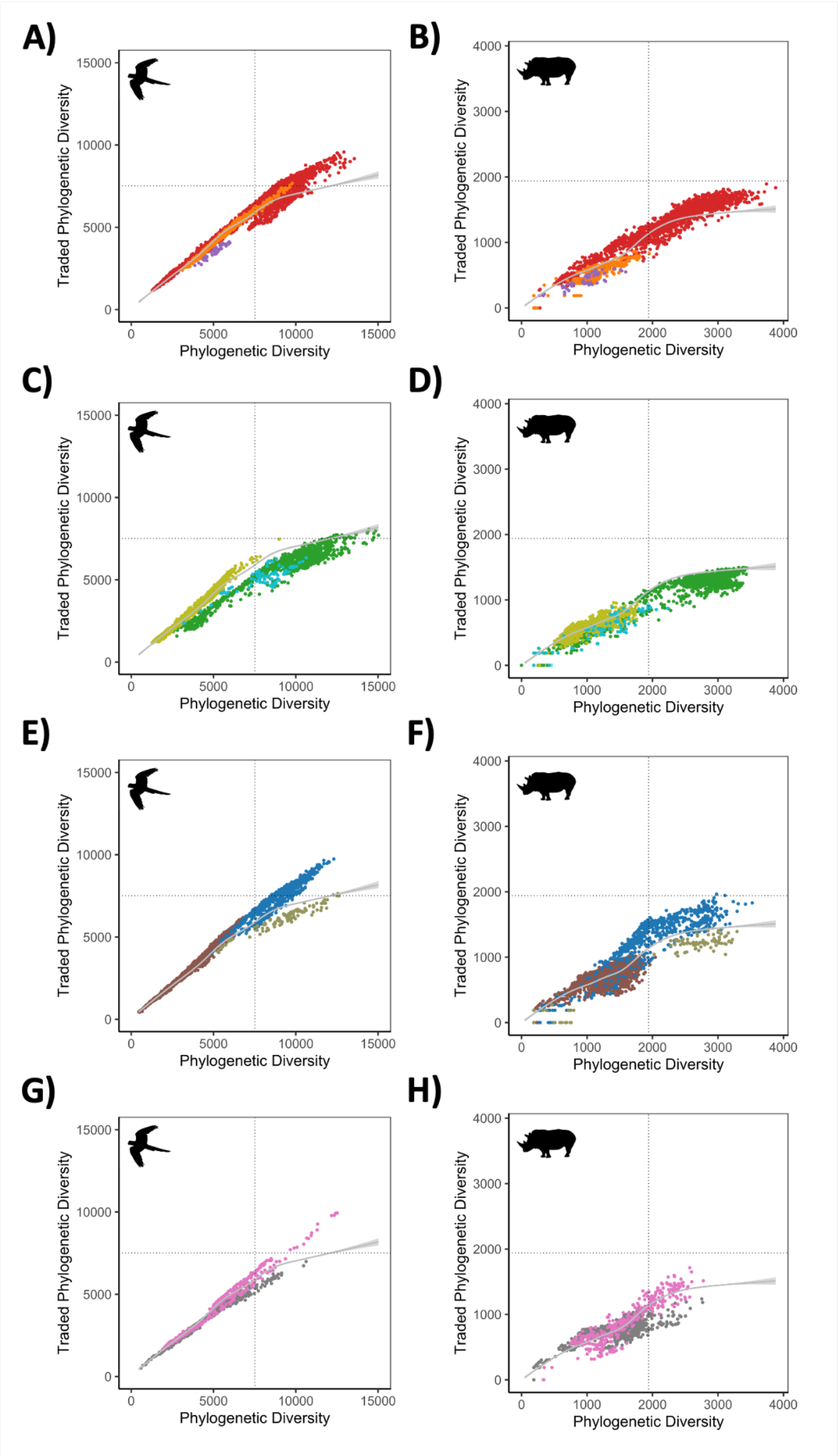

Figure S2. The relationship between traded PD and overall PD levels in each biogeographic realm. (A and B) Afrotropical, Australian and Madagascan realms. (C and D) Neotropical, Saharo-Arabian and Oceanian realms. (E and F) Oriental, Panamanian and Palearctic realms. (G and H) Nearctic and Sino-Japanese realms. The grey lines in scatterplots indicate a locally estimated scatterplot smoothing (LOESS) fit of the global trend.

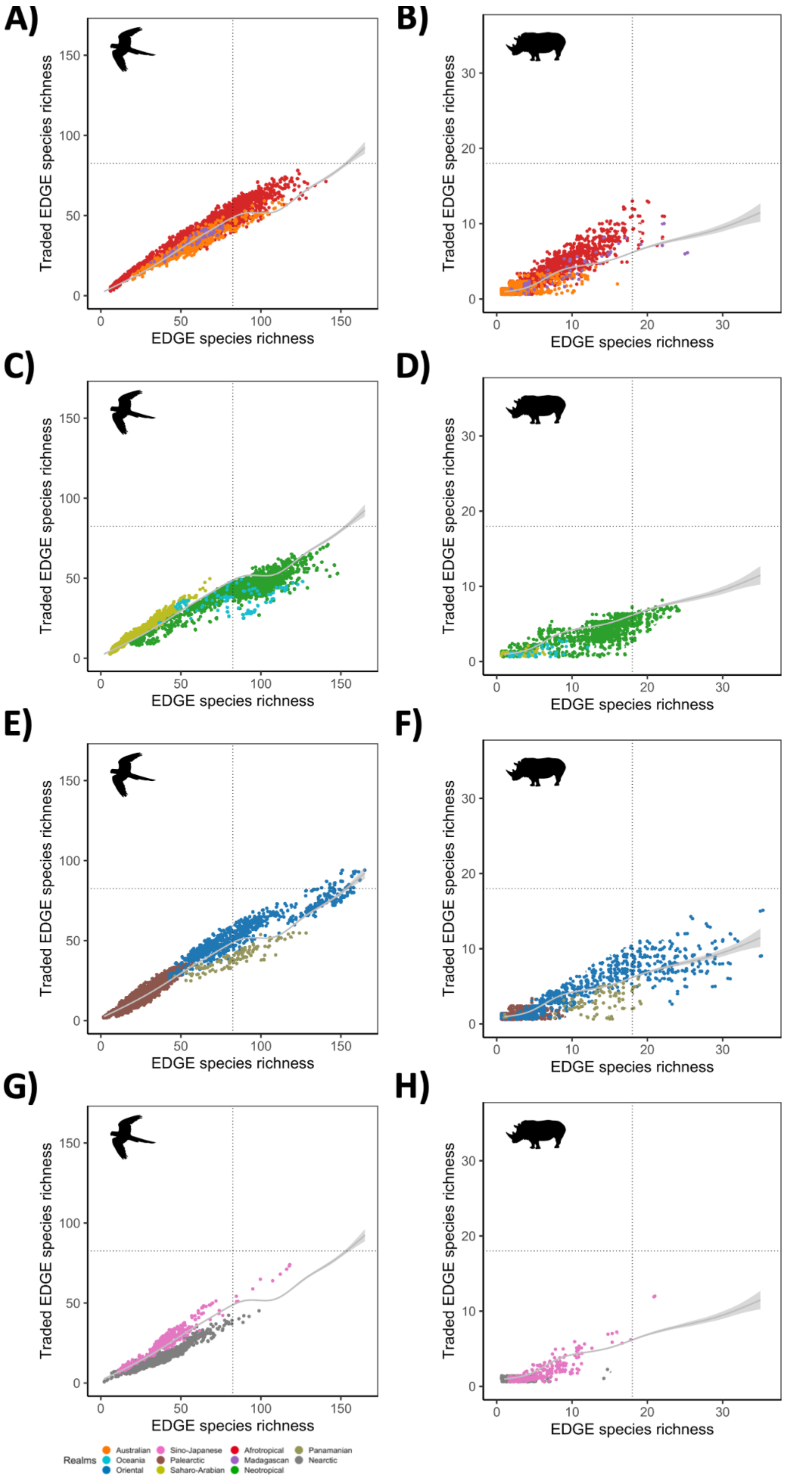

Figure S3. The relationship between the top 25% of traded EDGE species richness and the top 25% of overall species richness levels in each biogeographic realm. (A and B) Afrotropical, Australian and Madagascan realms. (C and D) Neotropical, Saharo-Arabian and Oceanian realms. (E and F) Oriental, Panamanian and Palearctic realms. (G and H) Nearctic and Sino-Japanese realms. The grey lines in scatterplots indicate a locally estimated scatterplot smoothing (LOESS) fit of the global trend.

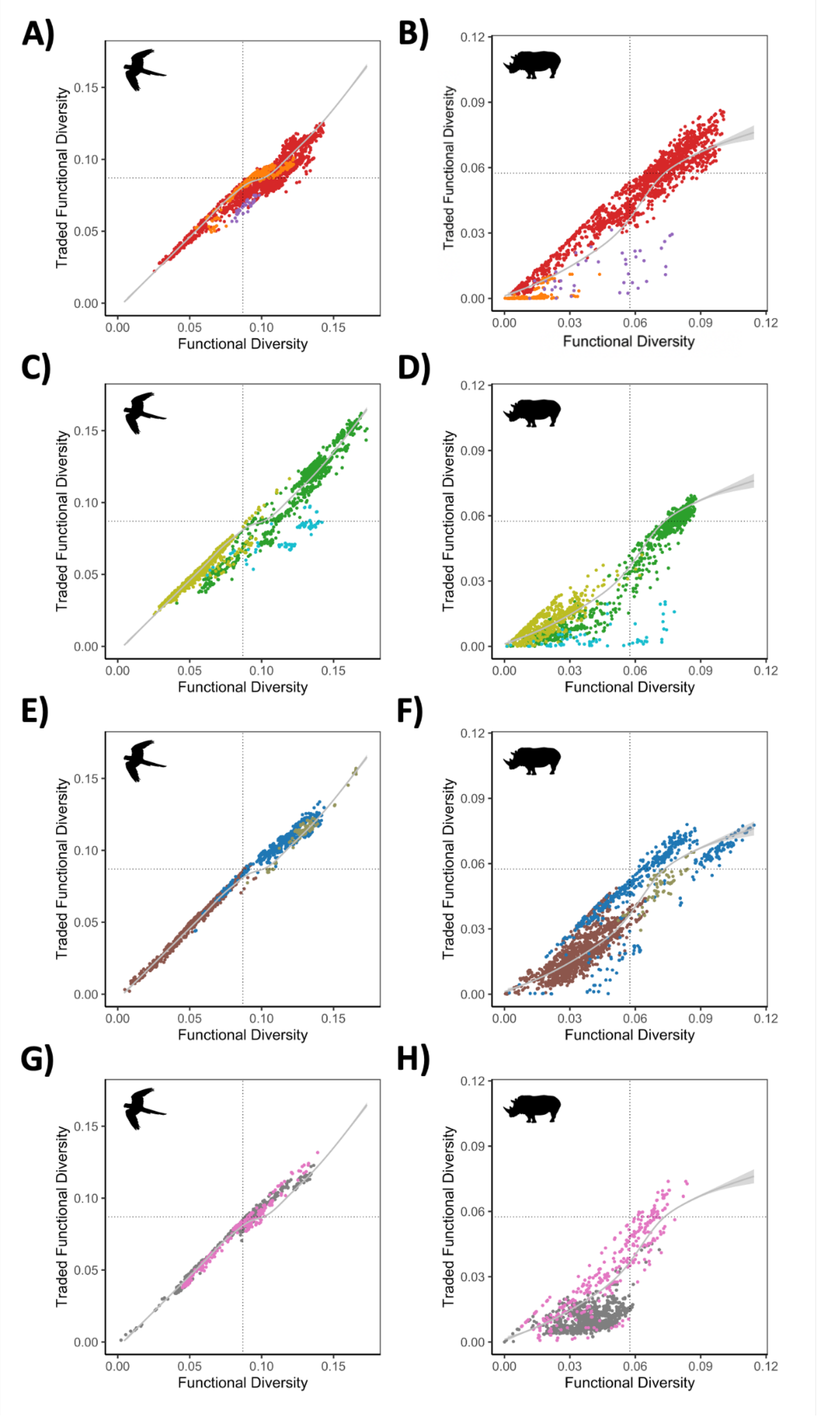

Figure S4. The relationship between traded FD and overall FD levels in each biogeographic realm. (A and B) Afrotropical, Australian and Madagascan realms. (C and D) Neotropical, Saharo-Arabian and Oceanian realms. (E and F) Oriental, Panamanian and Palearctic realms. (G and H) Nearctic and Sino-Japanese realms. The grey lines in scatterplots indicate a locally estimated scatterplot smoothing (LOESS) fit of the global trend.
