# Supplementary material for: Global hotspots of traded phylogenetic and functional diversity
Source: Nature. 2023 Jul 26;620(7973):351–7. doi: 10.1038/s41586-023-06371-3 (PMC10412452; doi:10.1038/s41586-023-06371-3)
Supplement: Supplementary file 2 — Reporting Summary [file 41586_2023_6371_MOESM2_ESM.pdf]

Corresponding author(s): David P Edwards

Last updated by author(s): Apr 21, 2023

Reporting Summary

Nature Portfolio wishes to improve the reproducibility of the work that we publish. This form provides structure for consistency and transparency in reporting. For further information on Nature Portfolio policies, see our Editorial Policies and the Editorial Policy Checklist.

Statistics

For all statistical analyses, confirm that the following items are present in the figure legend, table legend, main text, or Methods section.

n/a	Confirmed	
☐	☒	The exact sample size (*n*) for each experimental group/condition, given as a discrete number and unit of measurement
☒	☐	A statement on whether measurements were taken from distinct samples or whether the same sample was measured repeatedly
☐	☒	The statistical test(s) used AND whether they are one- or two-sided<br>*Only common tests should be described solely by name; describe more complex techniques in the Methods section.*
☐	☒	A description of all covariates tested
☐	☒	A description of any assumptions or corrections, such as tests of normality and adjustment for multiple comparisons
☐	☒	A full description of the statistical parameters including central tendency (e.g. means) or other basic estimates (e.g. regression coefficient) AND variation (e.g. standard deviation) or associated estimates of uncertainty (e.g. confidence intervals)
☐	☒	For null hypothesis testing, the test statistic (e.g. *F*, *t*, *r*) with confidence intervals, effect sizes, degrees of freedom and *P* value noted<br>*Give P values as exact values whenever suitable.*
☐	☒	For Bayesian analysis, information on the choice of priors and Markov chain Monte Carlo settings
☐	☒	For hierarchical and complex designs, identification of the appropriate level for tests and full reporting of outcomes
☐	☒	Estimates of effect sizes (e.g. Cohen's *d*, Pearson's *r*), indicating how they were calculated

Our web collection on statistics for biologists contains articles on many of the points above.

Software and code

Policy information about availability of computer code

Data collection	All data used is sourced from existing published studies: Trade data from Scheffers et al. 2019 Science, traits data from EltonTraits 1.0 see here https://opentraits.org/datasets/elton-traits.html and all phylogeny data from https://vertlife.org/data.
Data analysis	All analyses where undertaken in R version 4.2.1. Key software packages used included "tidyverse" 1.3.2, "ape" 5.4-1, "mFD" 1.0.1, "brms" 2.18.0, "tidybayes" 3.0.2, "betareg" 3.1-4, "rphylopars" 0.3.2 and "bayestestR" 0.11.5.

For manuscripts utilizing custom algorithms or software that are central to the research but not yet described in published literature, software must be made available to editors and reviewers. We strongly encourage code deposition in a community repository (e.g. GitHub). See the Nature Portfolio guidelines for submitting code & software for further information.

Data

Policy information about availability of data

All manuscripts must include a data availability statement. This statement should provide the following information, where applicable:

- Accession codes, unique identifiers, or web links for publicly available datasets
- A description of any restrictions on data availability
- For clinical datasets or third party data, please ensure that the statement adheres to our policy

All data used is available upon publication in a publicly accessible repository.

Human research participants

Policy information about studies involving human research participants and Sex and Gender in Research.

Reporting on sex and gender	NA
Population characteristics	NA
Recruitment	NA
Ethics oversight	NA

Note that full information on the approval of the study protocol must also be provided in the manuscript.

Field-specific reporting

Please select the one below that is the best fit for your research. If you are not sure, read the appropriate sections before making your selection.

☐ Life sciences ☐ Behavioural & social sciences ☒ Ecological, evolutionary & environmental sciences

For a reference copy of the document with all sections, see nature.com/documents/nr-reporting-summary-flat.pdf

Ecological, evolutionary & environmental sciences study design

All studies must disclose on these points even when the disclosure is negative.

Study description	An analysis of the functional and phylogenetic diversity of species in trade. Raw and standardized metrics are calculated and visualized globally. We further assess the distribution of this using geographic covariates (realm) see Methods for more details. We also examine correlates of species presence in trade using the same functional traits used to calculate functional diversity.
Research sample	All data used is sourced from existing published studies: Trade data from Scheffers et al. 2019 Science, traits data from EltonTraits 1.0 see here https://opentraits.org/datasets/elton-traits.html and all phylogeny data from https://vertlife.org/data. All amphibian, bird, mammal and reptiles species were included except in instances where functional data was absent and no resolved phylogeny of that species was available to impute species traits.
Sampling strategy	NA
Data collection	LH collated the data from existing published data sources.
Timing and spatial scale	NA
Data exclusions	No data was excluded except in some sensitivity analyses where we excluded species with occupancy across multiple realms. Similarly some species could not be included as we lacked functional or phylogeny data. See methods for full details.
Reproducibility	NA - no experiments were undertaken. However, we report a number of supplementary analyses that where designed to check the robustness of our results. These include presenting raw PD, ED and FD metrics while also calculating the standardized effect size after incorporating species richness and running additional analyses on subsets of the species pool to exclude species with occupancy across multiple realms. See methods for more detail.
Randomization	NA
Blinding	NA

Did the study involve field work? ☐ Yes ☒ No

Reporting for specific materials, systems and methods

We require information from authors about some types of materials, experimental systems and methods used in many studies. Here, indicate whether each material, system or method listed is relevant to your study. If you are not sure if a list item applies to your research, read the appropriate section before selecting a response.

Materials & experimental systems

n/a	Involved in the study
☒	☐ Antibodies
☒	☐ Eukaryotic cell lines
☒	☐ Palaeontology and archaeology
☒	☐ Animals and other organisms
☒	☐ Clinical data
☒	☐ Dual use research of concern

Methods

n/a	Involved in the study
☒	☐ ChIP-seq
☒	☐ Flow cytometry
☒	☐ MRI-based neuroimaging
